# Antibacterial and Antifungal Sesquiterpenoids: Chemistry, Resource, and Activity

**DOI:** 10.3390/biom12091271

**Published:** 2022-09-09

**Authors:** Hang-Ying Li, Wen-Qian Yang, Xin-Zhu Zhou, Fei Shao, Tong Shen, Hui-Ying Guan, Jie Zheng, Li-Ming Zhang

**Affiliations:** 1 College of Pharmacy, Ningxia Medical University, Yinchuan 750004, China; 2 Ningxia Research Center of Modern Hui Medicine Engineering and Technology, Ningxia Medical University, Yinchuan 750004, China; 3 Key Laboratory of Ningxia Ethnomedicine Modernization, Ministry of Education, Ningxia Medical University, Yinchuan 750004, China

**Keywords:** sesquiterpenoids, chemical structures, antibacterial activity, antifungal activity, mechanism, structure–activity relationship

## Abstract

Infectious diseases caused by bacteria and fungi are threatening human health all over the world. It is an increasingly serious problem that the efficacies of some antibacterial and antifungal agents have been weakened by the drug resistance of some bacteria and fungi, which makes a great need for new antibiotics. Sesquiterpenoids, with abundant structural skeleton types and a wide range of bioactivities, are considered as good candidates to be antibacterial and antifungal agents. In the past decades, many sesquiterpenoids were isolated from plants and fungi that exhibited good antibacterial and antifungal activities. In this review, the names, source, structures, antibacterial and antifungal degrees, and mechanisms of sesquiterpenoids with antibacterial and antifungal activity from 2012 to 2022 are summarized, and the structure-activity relationship of these sesquiterpenoids against bacteria and fungi is also discussed.

## 1. Introduction

The infections caused by drug-resistant bacteria and drug-resistant fungi are increasing across the world, and the threat of untreatable infections has been looming since the 21st century [1]. About 4.95 million people died from diseases related to antibiotic-resistant bacteria in 2019, and 1.27 million deaths were directly caused by antibiotic-resistant bacteria, which indicated that drug-resistant infections killed more people than HIV/AIDS (864,000 deaths) or malaria (643,000 deaths) [2]. Fungi also led to life-threatening systemic infections, with a mortality of over 1.6 million, which is three times more than malaria, resulting in the widespread use of antifungal agents [3]. The efficacy of the limited systemic antifungal drugs was counteracted by fungal attributes and host- and drug-related factors. Furthermore, some fungal pathogens showed notable rates of antifungal resistance, including *Candida, Aspergillus, Cryptococcus*, and *Pneumocystis* [4]. Therefore, it is a challenge that antibiotic resistance is not easy to overcome, requiring the development of newer antibacterial and antifungal drugs [3].

Natural products, which have rich resources and great bioactivities, play an important role in the discovery of new drugs [5]. A total of 60% of the small molecule drugs marketed from 1981 to 2019 arose from natural products or synthetic molecules based on natural product pharmacophores [6]. Sesquiterpenoids are the most abundant natural products, with various activities and excellent prospects in drug development. For example, qinghaosu (artemisinin), a sesquiterpenoid lactone from *Artemisia annua* discovered by Tu Youyou, has already reached the market as an antimalarial drug [7,8]. Santonin, a sesquiterpenoid compound, has been marketed as an anthelmintic and used for a long time against ascaris infection with a remarkable curative effect which exhibited certain inhibitory effects on bacteria and fungi [9,10]. There are some other sesquiterpenoids compounds, such as parthenolide, alantolactone, bilobalide, coriaria lactone, and cycloeudesmol, that have already been commercialized [11,12,13,14,15]. Aiming to look for better antimicrobial leads, the names, structures, source, antibacterial and antifungal degree, and mechanisms of natural sesquiterpenoids with antibacterial activity from 2012 to 2022 are systematically and completely summarized in this review (Tables S1 and S2). The structure–activity relationship of sesquiterpenoids with significant antibacterial and antifungal activity is discussed as well. This review will provide support for the use of sesquiterpenoids as potential antibacterial agents in the future.

## 2. Sesquiterpenoids Types

**Figure 1:**
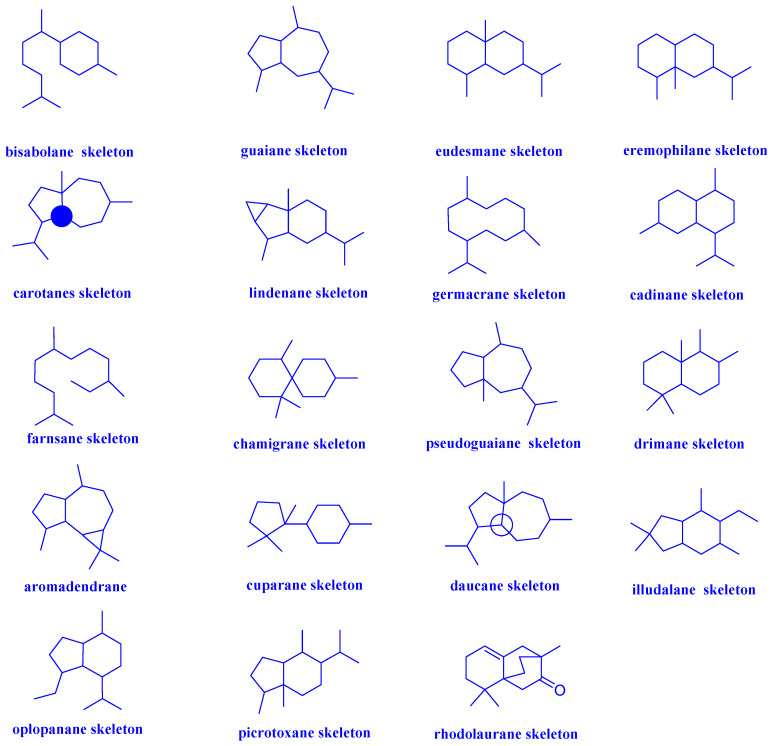
Different types of sesquiterpenoid skeletons.

**Figure 2:**
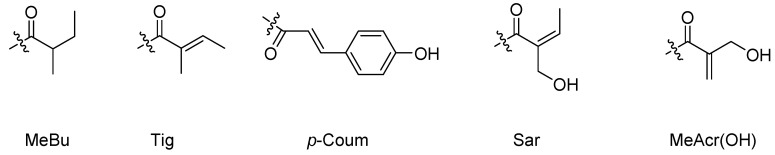
Groups in shorthand.

Sesquiterpenoids, C_15_ compounds composed of three isoprene units, are one family of structurally diverse natural products [16]. Today, thousands of sesquiterpenoids have been discovered with more than 100 skeleton types. Regarding their carbon skeletons, sesquiterpenoids with antibacterial and antifungal activity mainly include bisabolane, guaiane, eudesmane, eremophilane, carotane, lindenane, germacrane, cadinane, farnsane, chamigrane, pseudoguaiane, drimane, aromadendrane, cuparane, daucane, illudalane, oplopanane, picrotoxane, rhodolaurane and other types, which were showed in Figure 1. The groups used shorthands mentioned in compounds structure were demonstrated in Figure 2.

### 2.1. Bisabolanes

**Figure 3:**
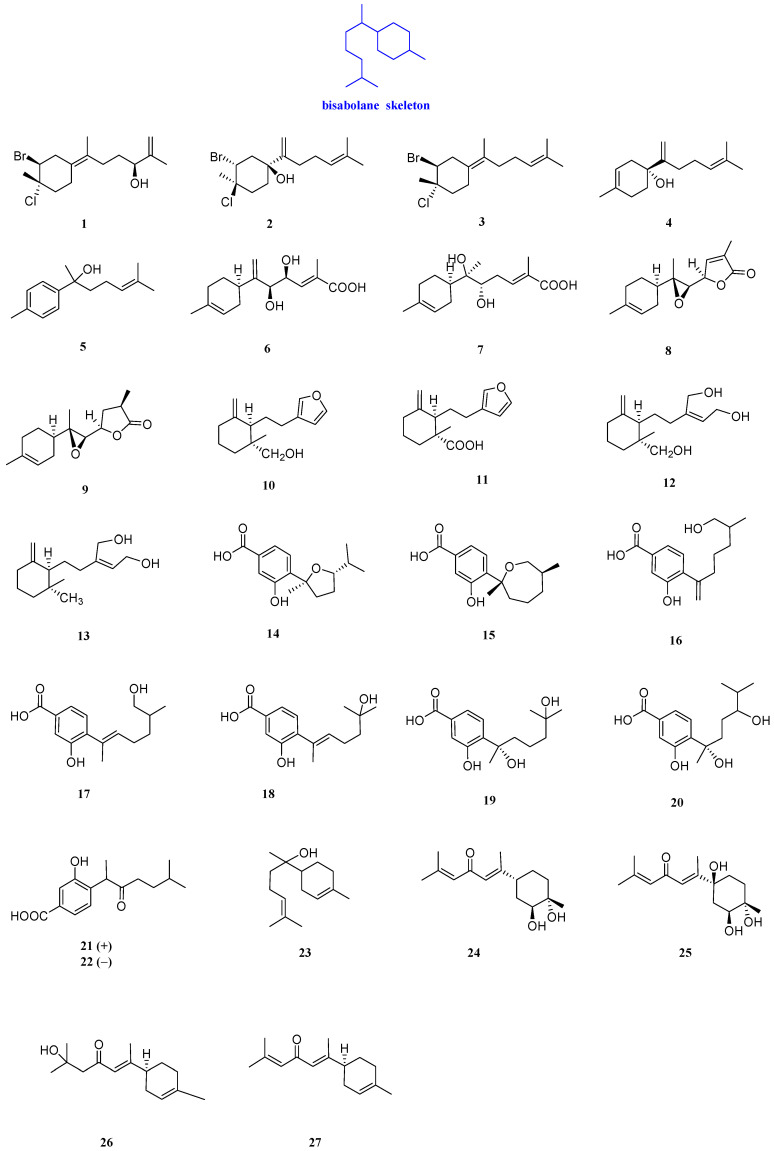
Structures of bisabolanes-type sesquiterpenoids **1**–**27**.

Laurecomposin A, laurecomposin B, preintricatol, helianthol B, and gossonorol (**1**–**5**, Figure 3) were isolated from the red alga *Laurencia tristicha.* The complete 1H and 13C NMR assignments of compound **1** were made by a combination of 1H, 13C, DEPT, 1He1H COSY, HSQC, HMBC, and ROESY experiments and the absolute configuration was established by the modified Mosher’s method. It was confirmed that compound **1** had activity against *Staphylococcus aureus* (*S. aureus*) and *Candida albicans* (*C. albicans*) SC5314 with MIC values of 26.8 and 16 µg/mL, respectively. Compound **1** also had an obvious inhibitory effect on *Microsporum gypseum* (*M. gypseum*), and the MIC value was 4.0 µg/mL. In the antifungal and antibacterial assays, compound **2** exhibited significant inhibitory activities against *M. gypseum* (Cmccfmza) and moderate activities towards *S. aureus*, with MIC values of 8 and 15.4 μg/mL, respectively. Compound **3** had inhibitory activity against *C. albicans* SC5314, *S. aureus*, and *M. gypseum*, with MIC values of 32, 13.6, and 8 μg/mL. In addition, compounds **4** and **5** had inhibitory activity against *S. aureus* and *M. gypseum*, with MIC values of 4−54 μg/mL [17].

Six sesquiterpenoids with antibacterial activity were isolated from a basidiomycete collected in Mount Elgon Natural Reserve, named elgonene C, elgonene D, elgonene G, elgonene H, elgonene I, elgonene J, elgonene K, and elgonene L (**6**–**13**, Figure 3). To determine the stereochemistry of elgonene J, elgonene K, and elgonene L, CD spectra of these three compounds were measured. Compounds **6**–**13** exhibited an inhibitory effect on *M. hiemalis* DSM 2656*,* with MIC values of 25 to 100 µg/mL. Compounds **6**, **8**/**9**, and **13** had weak inhibitory activity against *S. aureus* DSM 346 with a MIC value of 100 μg/mL. Compounds **8**–**11** and **13** (**8**/**9** were tested as an inseparable mixture) showed weak antimicrobial activity against *Bacillus subtilis* (*B. subtilis*) DSM 10, with MIC values of 100, 100, 75, 75, and 100 μg/mL, respectively. Compounds **8**/**9** and **13** demonstrated weak activities against *Micrococcus luteus* (*M. luteus*) DSM 1790 (same MIC value of 100 μg/mL) and moderate activity against *M. hiemalis* DSM 2656 (MIC values of 100 and 25 μg/mL, respectively). No activity against Gram-negative bacteria or yeast was observed. Compounds **6**–**9** and **11**–**13** showed antibacterial activity against *M. hiemalis* DSM 2656, with MIC values of 100, 50, 100, 50, 50, 25, and 50 μg/mL [18].

There were seven *bisabolane-type* sesquiterpenoids with antibacterial activity from the leaves of a Thai mangrove *Xylocarpus moluccensis*, named (7*S*,10*S*)-7,10-epoxysydonic acid, (7*R*,11*S*)-7,12-epoxysydonic acid, 7-deoxy-7,14-didehydro-12-hydroxysydonic acid, (*E*)-7- deoxy-7, 8-didehydro-12-hydroxysydonic acid, engyodontiumone I, (+)-hydroxysydonic acid, and (−)-(7*S*)-10-hydroxysydonic acid (**14**–**20**, Figure 3). The potent inhibitory activitiy of compounds **14**–**20**, for *S. aureus* ATCC 25923, was evaluated by liquid phase inhibition in 96-well microplates, and the IC_50_ values were determined to be at 31.5–41.9 µM [19].

(+)-Phomoterpene A and (−)-phomoterpene A (**21** and **22**, Figure 3), isolated from the endophytic fungus *Phomopsis prunorum* (F4-3), showed inhibitory activity against *X. citri* pv. *Phaseoli* var. *fuscans*, with MIC values of 31.2 and 62.4 µg/mL, respectively. Additionally, both of the compounds exhibited inhibitory against the plant pathogen *Pseudomonas syringae* pv (*P. syringae* pv). *Lachrymans* had the same MIC value of 15.6 µg/mL [20].

*α*-Bisabolol (**23**, Figure 3) is a low toxic monocyclic sesquiterpenoid alcohol from *Vanillosmopsis arborea* Barker. The IC_50_s of compound **23** against CA INCQS 40006, CK INCQS 40095, and CT INCQS 40042, different strains of *Candida*, were 8.92, 18.15, and 18.26 *µ*M, respectively [21]. (*E*)-(2*S*,3*S*,6*R*)-Atlantone-2,3-diol, (*E*)-(2*S*,3*S*,6*S*)-atlantone-2,3,6-triol, atlantolone, and (*E*)-*α*-atlantone (**24**–**27**, Figure 3) were isolated from *Cedrus deodara* Loud. Compound **24** had a weak inhibitory effect on *Aspergillus sydowii* (*A. sydowii*) and *Aspergillus parasiticus* (*A. parasiticus*), with MIC values of 6400 and 3200 µg/mL, respectively. Compound **25** was active against *Trichophyton rubrum* (*T. rubrum*), with a MIC value of 125 µg/mL, while compounds **26** and **27** exhibited an inhibitory effect on *Aspergillus niger* (*A. niger*)*, A. sydowii, A. parasiticus, A. ochraceous,* and *A. flavus,* with MIC values from 200 to 6400 µg/mL [22].

### 2.2. Guaianes

**Figure 4:**
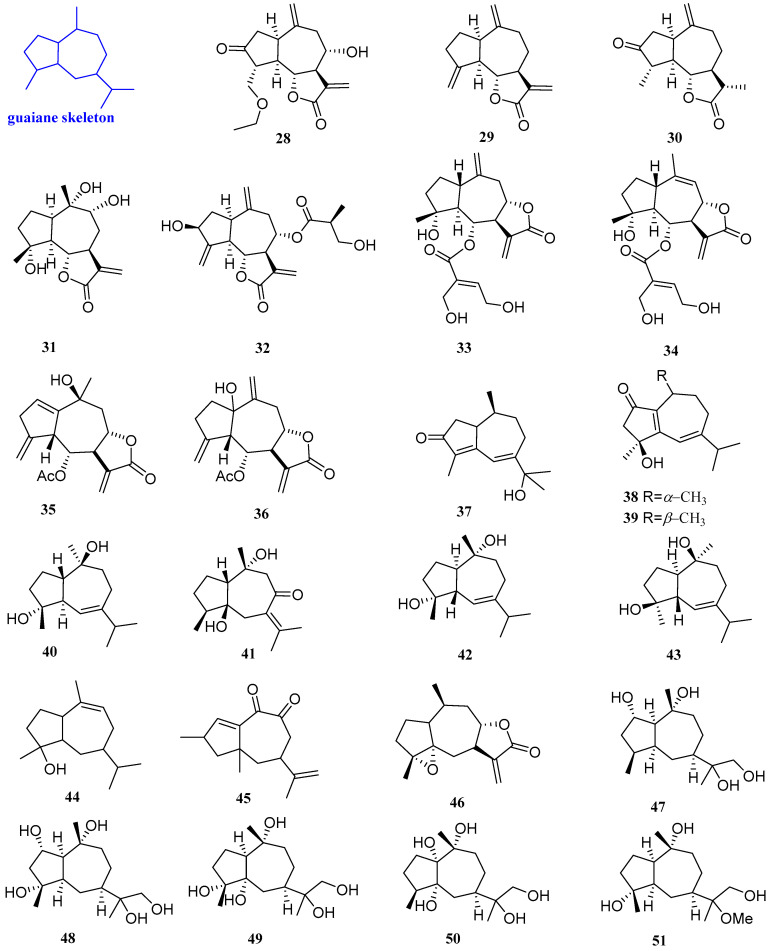
Structures of guaiane-type sesquiterpenoids **28**–**51**.

Three new sesquiterpenoids were isolated from *Artemisia vestita*, including artemivestinolide G, dehydrocostuslactone, and dihydroestafiatone (**28**−**30**, Figure 4). The antifungal test revealed that **29** had a good inhibitory effect on *Fusarium oxysporum* (*F. oxysporum*), with a MIC value of 256 mg/L. Compounds **28** and **30** had a certain antibacterial effect on *Botrytis cinerea* (*B. cinerea*), with a MIC value of 256 mg/L [23].

4*α*,9*α*,10*α*-Trihydroxyguaia-11(13)en-12,6*α*-olide (**31**, Figure 4) was collected from the leaves of the Saudi medicinal plant *Anvillea garcinii*. Compound **31** had antibacterial activity against *C. albicans*, *C. parapsilosis, S. aureus, B. licheniformis,* and *E. fergusonii*, with MIC values of 0.21*,* 0.25*,* 2.3, 2.3, and 5.7 µg/mL, respectively [24]. 8-*O*-[3’-Hydroxy-2’-methylpropionate] (**32**, Figure 4), extracted from the aerial parts of *Centaurea rhizantha*, had moderate antibacterial activity against *S. aureus*, and the MIC/MBC value was 500 µg/mL. [25].

6*α*-[4’,5’-Dihydroxytigloyloxy]-inuviscolide and 6*α*-[4’,5’-dihydroxytigloyloxy]-isoinuviscolide (**33** and **34**, Figure 4) were extracted from *Schkuhria pinnata* (Lam.) Kuntze ex thell. A micro broth dilution method was used to test the antibacterial activity of compounds **33** and **34**. The results showed that compounds **33** and **34** both had antibacterial activity against *Escherichia coli* (*E. coli*), *Pseudomonas aeruginosa* (*P. aeruginosa*), *Enterobacter faecalis* (*E. faecalis*)*,* and *S. aureus,* and the MIC values were 125, 46.88, 125, and 62.5 µg/mL [26].

6-Acetoxy-10-*β*-hydroguaiantrienolide and 6-acetoxy-1*α*-hydroguaiantrienolide (**35** and **36**, Figure 4) were isolated from *Cotula cinerea*. Compounds **35** and **36** had antibacterial activity against some strains of *Enterobacter faecalis*, EF-91804, EF-91823, EF-165, EF-91705, and ATCC29212, and the MICs were 150, 300, 300, 300, and 300µg/mL, respectively. [27]. Sootepdienone, jambolanins E, jambolanins F, and guaianediol (**37**–**40**, Figure 4) were isolated from the seeds of *Eugenia jambolana* fruit and exhibited inhibitory activity against *S. aureus*, with a diameter of the inhibitory zone of 9, 10, 10, and 9 mm, respectively [28].

Wenyujinin Q (41, Figure 4), isolated from a traditional Chinese medicine, *Curcuma wenyujin* dreg, had strong broad-spectrum antifungal activity against nine pathogenic fungi, including *Alternaria brassicicola* (*A. brassicicola*), *P. parasitica* var. *nicotianae*, *C. capsici*, *B. oryzae*, *D. medusaea Nitschke*, *C. paradoxa Moreau*, *E. turcicum*, *P. theae,* and *A. citri* (the MICs being 50, 100, 50, 50, 100, 50, 25, 25, and 100 µg/mL, respectively) [29]. 4*α*,10*α*-Dihydroxy-5*β*-*H*-guaja-6-ene (**42**, Figure 4) was isolated from *Cassia buds*. **42** had antibacterial activity against *C. albicans* and *S. aureus*, with inhibitory zones of 9 and 7.5 mm, respectively [30].

4*β*, 10*β*-Dihydroxy-1*α*H, 5*β*H-guaia-6-ene (**43**, Figure 4), obtained from the rhizome of *Alisma orientale*, could inhibit the activity of *B. subtilis* and possessed a MIC value of 50 µg/mL [31]. Two sesquiterpenoids named guai-9-en-4*β*-ol and 14,15-dinorguai-1,11-dien-9,10-dione (**44** and **45**, Figure 4) were isolated from the stem of *Syringa pinnatifolia* Hemsl. var. alashanensis. Compound **44** had strong inhibition against *E. coli, S. aureus, B. coagulas, Proteus vulgaris* (*P. vulgaris*)*, P. digitatum, F. oxysporum, and A. niger*, with inhibitory zones of 11.02, 13.41, 15.34, 9.67, 12.56, 11.64, and 13.20 mm, respectively. Compound **45** had good inhibitory activity against *E. coli, S. aureus, B. coagulas, P. vulgaris, P. digitatum, F. oxysporum, and A. niger*, with inhibitory zones of 15.34, 9.45, 12.01, 14.96, 12.34, 15.32, and 11.53 mm, respectively [32].

4*α*,5*α*-Epoxy-10*α*,14*H*-1-*epi*-inuviscolide (**46**, Figure 4) was isolated from *Carpesium macrocephalum*. The antibacterial experiments showed that **46** had certain inhibitory effects against *C. albicans* and the yeast-to-hyphae morphogenetic transition, with IC_50_ values of 38 and 106.5 µg/mL [33]. Five sesquiterpenoids were isolated from the Endophytic Fungus *Xylaria* sp. YM 311647 of *Azadirachta indica* A. Juss., identified as (1*S*,2*S*,4*S*,5*S*,7*R*,10*R*)-Guaiane-2,10,11,12-tetraol, (1*S*,2*S*,4*R*,5*R*,7*R*,10*R*)-Guaiane-2,4,10,11,12-pentaol, (1*S*,4*R*,5*S*,7*R*,10*R*)-Guaiane-4,5,10,11,12-pentaol, (1*R*,4*S*,5*R*,7*R*,10*R*)-Guaiane-1,5,10,11,12-pentaol, and (1*R*,4*R*,5*R*,7*R*,10*R*)-11-Methoxyguaiane-4,10,12-triol, respectively (**47**–**51**, Figure 4). The antifungal activity of compounds **47**–**51** was evaluated by the micro broth dilution method, which indicated that compounds **47**–**51** exhibited moderate or weak antifungal activities against *C. albicans*, *Pyricularia oryzae* (*P. oryzae*)*,* and *Hormodendrum compactum* (*H. compactum*), with MIC values in the range of 32–256 μg/mL. Compounds **47**–**51** had antibacterial activity against *A. niger*, with MIC values of 256, 64, 256, and 256 µg/mL [34].

### 2.3. Eudesmanes

**Figure 5:**
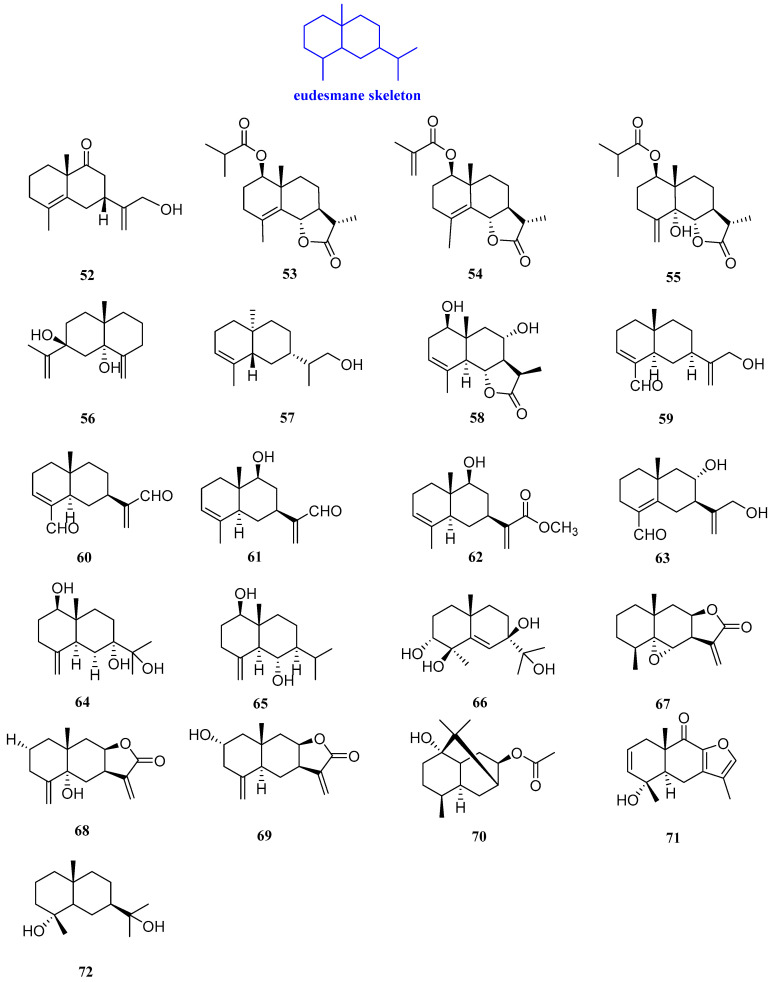
Structures of eudesmane-type sesquiterpenoids **52**–**72**.

Sutchuenin J (**52**, Figure 5) is a eudesmane*-type* sesquiterpenoid extracted from the EtOAc soluble fraction of the ethanolic extract of the stems and roots of *Thuja sutchuenensis*. Compound **52** displayed good antibacterial activities against *Bacillus cereus* (*B. cereus*) (ATCC 10876) and *Staphylococcus epidermidis* (*S. epidermidis*) (ATCC 12228), with the same MIC value of 25 µg/mL [35].

Artemivestinolides D–F (**53**–**55**, Figure 5) were isolated from *Artemisia vestita*, Compound **53** had a good inhibitory effect on *Pyricularia oryzae*, with a MIC value of 128 mg/L. Compounds **53** and **54** against *B. cinerea*, with the same MIC value of 256 mg/L. For *F. oxysporum*, compound **55** displayed antifungal activity, with a MIC value of 256 mg/L [23].

Eudesma-4(15),11-diene-5,7-diol (**56**, Figure 5) was extracted from *Laurencia obtusa* Lamouroux with antibacterial activity. Compound **56** possessed antifungal activity against *C. albicans* and *Candida tropicalis* (*C. tropicalis*), with MIC values of 8.27 and 10.13 µM, respectively. The antifungal activity of compound **56** was higher than amphotericin B (MICs = 4.63 and 5.27 µM, respectively) [36].

Eutyscoparin G (**57**, Figure 5) was isolated from the ethyl acetate extract of the endophytic fungus *Eutypella scoparia* SCBG-8. It was found that compound **57** could inhibit *S. aureus* and methicillin-resistant *S. aureus* with the same MIC value of 6.3 µg/mL [37]. 1*R*,8*S*-dihydroxy-11*R*,13-dihydrobalchanin (**58**, Figure 5) was extracted from the CH_2_Cl_2_ extract of *Artemisia Sieberi*. The agar diffusion technique was followed and the inhibition zone of **58** against *B. subtilis*, *S. aureus*, *E. coli*, *F. solani*, *P. aeruginosa*, *C.* *tropicalis,* and *F. solani* was 6–8 mm, which was more effective than thiophenicol, the positive control which was a broad-spectrum antibacterial antibiotic [38].

The antibacterial activities of (4*αβ*,7*β*,8*αβ*)-3,4,4*α*,5,6,7,8,8*α*-octahydro-7-[1-(hydroxymethyl)ethenyl]-4*α*-methylnaphthalene-1-car boxaldehyde, 12,15-dioxo-*α*-selinen, (5*S,*7*S,*9*S,*10*S)-*(+)-9-hydroxy-selina-3,11-dien-12-al, (5*S,*7*S,*9*S*,10*S*)-(+)-9-hydroxy-eudesma-3,11(13)-dien-12-methyl ester, and (7*S,*8*R*,10*S*)-(+)-8,12-dihydroxy-selina-4,11-dien-14-al (**59**–**63**, Figure 5), isolated from Chinese agarwood, were measured by inhibition zone diameters. All these compounds could inhibit *S.aureus*, with inhibitory zones of 9.12, 20.02, 12.90, 14.20, and 8.10 mm, respectively. Compounds **59**–**63** had antibacterial activity against *Ralstonia solanacearum* (*R. solanacearum*), with inhibitory zones of 8.98, 11.02, 18.20, and 10.15 mm, respectively, while 63 had no activity against *R. solanacearum* [39].

4(15)-Eudesmene-1*β*,7,11-triol, 1*β*,6*α*-dihydroxyeudesm-4(15)-ene, and cinnamosim B (**64**–**66**, Figure 5) are three eudesmane*-type* sesquiterpenoids isolated from *Cassia buds* which have antimicrobial activity against *C. albicans*, *S. aureus*, and *E. coli.* Compounds **64**–**66** selectively inhibited the proliferation of *C. albicans*, with inhibitory zones of 9, 11, and 10 mm, respectively. Compounds **65** and **66** could also inhibit the proliferation of *S. aureus*, with inhibitory zones of 11 and 9 mm, respectively. Compound **64** only showed inhibitory effects on *E. coli*, with an inhibitory zone of 8.5 mm [30].

Three germacrane-type sesquiterpenoids were extracted and isolated from the whole plant of *Carpesium macrocephalum*, named 5*α*-epoxyalantolactone, telekin, and ivalin (**67**–**69**, Figure 5). Compounds **68** and **69** inhibited biofilm formation of *C. albicans*, with IC_50_ values ranging from 15.4 to 36.0 μg/mL, and compound **67** inhibited the yeast-to-hyphae morphogenetic transition, with an IC_50_ value of 118.4 μg/mL [33].

8-Acetoxyl-pathchouli alcohol (**70**, Figure 5) was isolated from the roots of *Valeriana jatamansi* Jones, and its antibacterial activity was identified by the micro broth dilution method. The antibacterial assays revealed that compound **70** had a certain inhibitory effect on *S. aureus* and *P. aeruginosa*, with MIC values of 128 and 64 µg/mL, respectively [40].

A sesquiterpenoid lactone named chlojaponol B (**71**, Figure 5) was isolated from *Chloranthus japonicus*. Compound **71** displayed a certain antibacterial activity against *B. cinerea* and *S. sclerotiorum*, with inhibitory rates of 34.62% and 13.04% at the concentration of 50 μg/mL [41].

Cryptomeridiol (**72**, Figure 5), isolated from the seeds of *Eugenia jambolana* fruit, exhibited inhibitory activity against *S. aureus*, with a diameter of the inhibitory zone of 8 mm [28].

### 2.4. Eremophilanes

**Figure 6:**
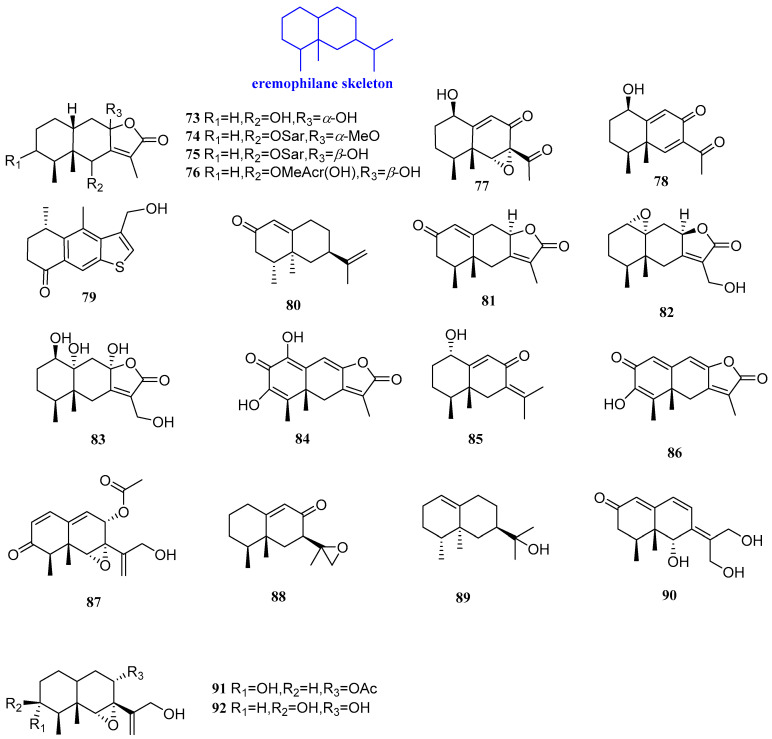
Structures of eremophilane-type sesquiterpenoids **73**–**92**.

Six sesquiterpenoids were isolated from *Ligularia sagitta*, named 1*β*,10*β*-epoxy-6*β*,8*α*-dihydroxyeremophil-7(11)-en-8*β*(12)-olide, sagittacin C, sagittacin D, 6*β*-(2’-hydroxymethylacryloyloxy)-1*β*,10*β*-epoxy-8*β*-hydroxyeremophila-7(11)-en-8*α*(12)-olide, sagittacin E, and 1*β*-hydroxy-6,9-dien-8-oxoeremophil-11-nor-11-ketone (**73**−**78**, Figure 6). It was confirmed that compounds **73**−**76** could inhibit *B. cereus*, *S. aureus*, *B. subtilis*, *E. coli*, and *Erwinia carotovora* (*E. carotovora*), with MIC values ranging from 7.25 to 125 µg/mL. Compound **78** exhibited moderate activity against *E. coli* and *E. carotovora*, with MIC values of 31.25 µg/mL and 62.5 µg/mL, respectively, and compound **78** showed only moderate inhibitory effects against *E. coli*, with a MIC value of 31.25 µg/mL [42].

Leptosphin A (**79**, Figure 6) was purified from the solid fermentation cultures of the endophytic fungus *Leptosphaeria* sp. XL026, isolated from the leaves of *Panax notoginseng*. The antibacterial and antifungal activities of compound **79** were tested. The results showed that compound **79** had antifungal activity against *Fusarium graminearum* (*F. graminearum*), *S. sclerotiorum*, *V. dahliae* Kleb, *B. carbonum* Wilson, *P. parasitica* Dastur, *A. alternata (Fries)* Keissler, and *B. cinerea* Pers, with the MIC value range of 25–100 µg/mL. Furthermore, compound **79** also showed medium antibacterial activity against *Micrococcus lysodeikticus* (*M. lysodeikticus*), *B. cereus*, *S. aureus*, *S. typhimurium*, and *E. aerogenes*, with MIC values of 50, 25, 100, 100, and 50 µg/mL, respectively [43]. Nootkatone (**80**, Figure 6) exists in grapefruit and has a variety of pharmacological effects. Researchers found that 80 had an inhibitory effect at the concentrations of 1 and 0.5 mM, respectively, for *L. monocytogenes* and *C. diphtheriae* [44].

Xylareremophil and eremophilane mairetolides B and G (**81**–**83**, Figure 6) were isolated from the endophytic fungus *Xylaria* sp. GDG-102, cultured from *Sophora tonkinensis.* For *P. vulgaris* and *Micrococcus luteum* (*M. luteum*), **81** displayed moderate activity, with the same MIC value of 25 µg/mL, and the MIC values of **83** were 25 μg/mL and 50 μg/mL, respectively. Compound **81** was found to be active against *M. luteum* with a MIC value of 50 μg/mL. Compounds **81**–**83** showed inhibitory effects on both *B. subtilis* and *M. lysodeikticus*, with a same MIC value of 100 µg/mL [45].

Rhizoperemophilane K, 1*α*-hydroxyhydroisofukinon, and 2-oxo-3-hydroxy-eremophila-1 (10),3,7 (11),8-tetraen-8,12-olide (**84**–**86**, Figure 6) were isolated from *Rhizopycnis vagum*. Compounds **84**–**86** had an inhibitory effect on four kinds of bacteria, including *P. lachrymans, R. solanacearum, S. haemolyticus,* and *X. vesicatoria*, with MIC values of 32–128 µg/mL [46]. 8*α*-Acetoxyphomadecalin C (**87**, Figure 6) was isolated from the endophyte *Microdiplodia* sp. WGHS5 and was evaluated for antifungal and antibacterial activity. The results revealed that 87 had equivalently effective antifungal activity against *B. cinerea* and *F. graminearum* at 100 μg/mL. [47].

7*α*H-9(10)-Ene-11,12-epoxy-8-oxoeremophilane and valerianol (**88** and **89**, Figure 6), were isolated from Chinese agarwood originating from *Aquilaria sinensis* (Lour.) Gilg. Compound **88** could inhibit *S. aureus* and *R. solanacearum*, with an inhibitory area of 12.35 and 16.90 mm, and compound **89** could inhibit *S. aureus* and *R. solanacearum*, with an inhibitory area of 10.10 and 8.86 mm [48].

Phomadecalin F, 8*α*-monoacetoxyphomadecalin D, and 3-*epi*-phomadecalin D (**90**−**92**, Figure 6) were isolated from the endophyte *Microdiplodia* sp. TT-12. Compounds **91** and **92** showed moderate antimicrobial activity against *P. aeruginosa* ATCC 15442 and *S. aureus* NBRC 13276, with an inhibitory area of 10–13 mm. Compound **90** could inhibit *P. aeruginosa* ATCC 15442, with an inhibitory zone of 8 mm [49].

### 2.5. Carotanes

**Figure 7:**
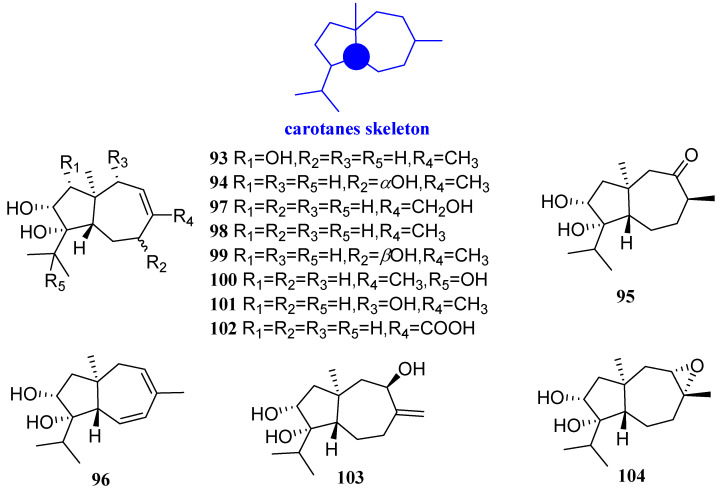
Structures of carotane-type sesquiterpenoids **93**–**104**.

Trichocarotins I–M, CAF-603, 7*β*-hydroxy CAF-603, trichocarotins E–H, trichocarane A (**93**–**104**, Figure 7) were found in the endophytic fungus *Trichoderma virens* QA-8 in the inner root tissue of mugwort leaves. The antibacterial activities of these compounds were assayed against human pathogens *E. coli* EMBLC-1 and *M. luteus* QDIO-3. Each of the compounds showed an inhibitory activity against *E. coli*, with the MIC values ranging from 0.5 to 32 µg/mL, and the activity of compounds **95**–**100** and **103** against *E. coli*, with the same MIC value of 0.5 µg/mL, which was as active as that of the positive control (chloramphenicol, MIC = 0.5 µg/mL). In addition, compounds **94**, **97**–**99**, **103**, and **104** showed inhibitory activity against *M. luteus*, with the MIC values ranging from 0.5 to 32 µg/mL. Compound **99** showed potent activity against *M. luteus*, with MIC values of 0.5 µg/mL, which was stronger than that of chloramphenicol (MIC = 1 µg/mL) [50].

### 2.6. Lindenanes

**Figure 8:**
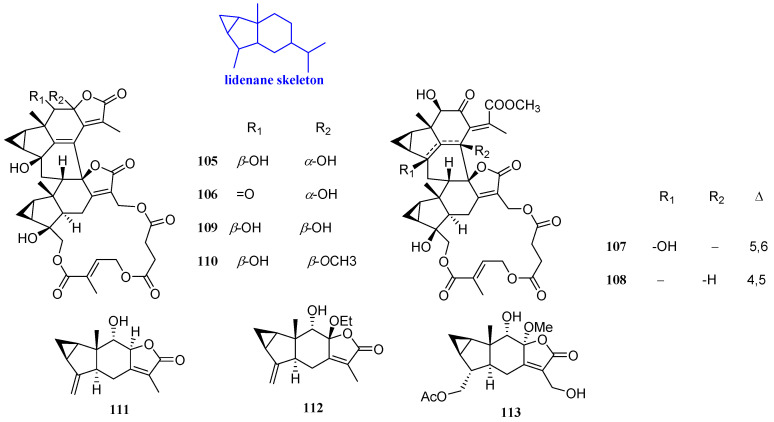
Structures of lindenane-type sesquiterpenoids **105**–**113**.

Six sesquiterpenoids, named henriol A, spicachlorantin A, chloramultilide A, shizukaol B, tianmushanol, and 8-*O*-methyltianmushanol (**105**–**110**, Figure 8), with antibacterial effects were isolated from the roots of *Chloranthus angustifolius*. Their antifungal activities were studied by microdilution method. Compounds **105**–**110** could inhibit the activity of *C. albican*s, and the MIC values were 4 to 8 µg/mL [51].

Three sesquiterpenoid lactones, named chlojaponilactones G-I (**111**–**113**, Figure 8), were isolated from *Chloranthus japonicus*. Compounds **111**–**113** displayed a certain antibacterial activity against *B. cinerea* and *S. sclerotiorum*, with inhibitory rates of 7.69% to 82.61% at the concentration of 50 μg/mL [41].

### 2.7. Germacranes

**Figure 9:**
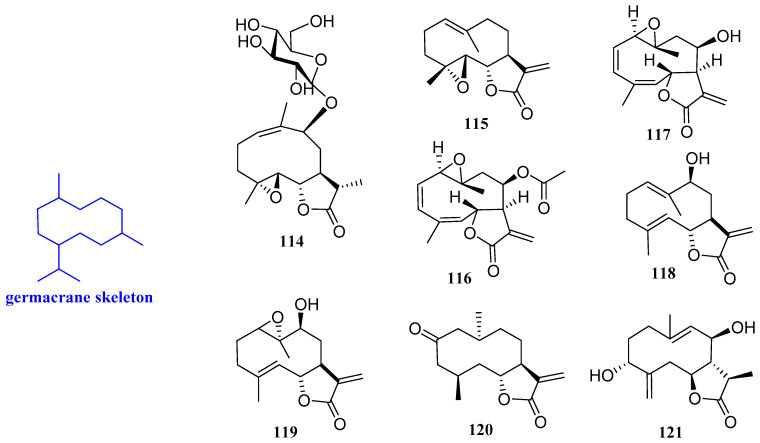
Structures of germacrane-type sesquiterpenoids **114**–**121**.

9*β*-Hydroxyparthenolide-9-*O*-*β*-D-glucopyranoside (**114**, Figure 9) was obtained from the leaves of the Saudi medicinal plant *Anvillea garcinii*. Compound **114** showed an inhibitory activity against human pathogenic fungi, which was about 80% at 50 μg/mL against *C*. *albicans* and *C. parapsilosis*, with MIC values of 0.26 μg/mL and 0.31 μg/mL, respectively. In addition, compound **114** inhibited *S*. *aureus*, *B. licheniformis,* and *E. fergusonii*, with MIC values of 3.4, 3.1, and 6.3 µg/mL, respectively [24].

Parthenolide (**115**, Figure 9) was isolated from Asteraceae and Magnoliaceae and is effective against various plant-pathogenic pathogens. The antibacterial assays revealed that compound **115** had a good inhibitory effect on *Erwinia amylovora* (*E. amylovora*) and *Corynebacterium fascians* (*C. fascians*), with the same MIC value of 20 mg/L. In addition, parthenolide is also effective against *V. mali*, *A. brassicicola*, and *P. piricola*, with EC_50_ values of 5, 2, and 5 mg/L, respectively [52].

Incomptine A and incompetine B (**116** and **117**, Figure 9) showed antibacterial activity against *Vibrio cholerae* (*V. cholerae*), with MIC values of 0.15 mg/mL and 0.05 mg/mL, respectively. The antibacterial activity of compounds **116** and **117** was better than chloramphenicol, which was used as positive control. This result suggested that **116** and **117** may be potential antibiotics for chloramphenicol-resistant bacteria, especially for *V. cholerae* [53].

Haagenolide and 1,10-epoxyhaagenolide (**118** and **119**, Figure 9) are two germacrane-type sesquiterpenoids isolated from the dichloromethane extract obtained from the aerial parts of *Cotula cinerea*. The absolute configuration was assigned by applying the advanced Mosher’s method to haagenolide and by X-ray diffraction analysis to 1,10-epoxyhaagenolide. *E. faecalis* *EF-91804*, *E. faecalis* *EF-91823*, *E. faecalis* *EF-165, and E. faecalis EF-91705* *were* four clinical bacteria isolated from *E. faecalis* and *used to* evaluate the antimicrobial activity of compounds **118** and 119. The results indicated that compound 118 could act against all mentioned *E. faecalis* above, with the same MIC value of 300 µg/mL. Compound **119** inhibited *EF-91804*, *EF-91823*, and *EF-165*, with the same MIC value of 300 µg/mL*, while it only inhibited* EF*-91705* with a MIC value of 150 µg/mL. Therefore, compounds **118** and **119** can be studied as new antibiotics [27].

Gracilone (**120**, Figure 9) was isolated from the methanol extract of *Tanacetum gracile.* Compound **120** showed moderate antibacterial activity against *S. aureus*, *B. subtilis*, *E. coli*, and *P. aeruginosa*, with diameters of the growth inhibition zones of 6.70, 14.3, 14.4, and 17.3 mm, respectively, determined by the agar disc diffusion method (50 μg/disk) [54].

3*R*,8*R*-Dihydroxygermacr-4(15),9(10)-dien-6*S*,7*S*,11*R*H,12,6-olide (**121**, Figure 9) is a new sesquiterpenoid lactone extracted from *Artemisia sieberi* with moderate antibacterial activity against *B*. *subtilis, S*. *aureus*, *E. coli,* and *P. aeruginosa*, with an inhibitory area of 6–8 mm [38].

### 2.8. Cadinanes

**Figure 10:**
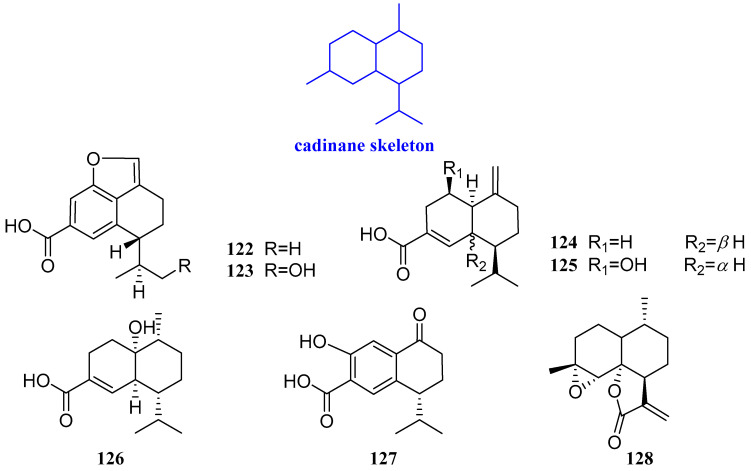
Structures of cadinane-type sesquiterpenoids **122**−**128**.

Trichocadinins B-G (**122**–**127**, Figure 10) were extracted from *Trichoderma virens* QA-8, an endophytic fungus obtained from the fresh inner tissue of the medicinal plant *Artemisia argyi*. The antimicrobial activities of compounds **122**–**127** were evaluated against one human pathogen (*E. coli* EMBLC-1), 10 marine-derived aquatic bacteria (*A. hydrophilia* QDIO-1, *E. tarda* QDIO-2, *E. ictarda* QDIO-10, *M. luteus* QDIO-3, *P. aeruginosa* QDIO-4, *V. alginolyticus* QDIO-5, *Vibrio anguillarum* (*V. anguillarum*) QDIO-6, *Vibrio harveyi* (*V. harveyi*) QDIO-7, *V. parahemolyticus* QDIO-8, and *V. vulnificus* QDIO-9), and 15 plant-pathogenic fungi (*A. solani* QDAU-14, *B. sorokiniana* QDAU-7, *C. cornigerum* QDAU-8, *C. diplodiella* QDAU-19, *Colletotrichum gloeosporioides* (*C. gloeosporioides*) *Penz* QDAU-9, *F. graminearum* QDAU-10, *F. oxysporum f. sp. cucumebrium* QDAU-16, *F. oxysporum f. sp. momordicae* QDAU-17, *F. oxysporum f. sp. radicis lycopersici* QDAU-5, *F. solani* QDAU-15, *G. cingulate* QDAU-2, *H. maydis* QDAU-18, *P. digitatum* QDAU-11, *P. piricola Nose* QDAU-12, and *V. mali* QDAU-13). Chloramphenicol and amphotericin B were used as the positive control against bacteria and fungi, respectively. Compounds **122**–**127** showed activity against *Fusarium oxysporum* f.sp. cucumebrium, with MIC values ranging from 1 to 64 μg/mL. Compound **127** had activity against aquatic pathogens *E. tarda* and *V. anguillarum*, with MIC values of 1 and 2 μg/mL, respectively, compared with that of the positive control chloramphenicol. Compound 122 exhibited inhibitory activity against the 12 test fungi (except *V. anguillarum*), with MIC values ranging from 1 to 64 μg/mL [55].

Arteannuin B (**128**, Figure 10) was isolated from *Leonurus japonicus* with a significant inhibitory effect on *E. coli* and *E. aerogenes* with the MIC values of 25 μg/mL and 50 μg/mL, respectively, by assaying the micro-dilution method [56].

### 2.9. Farnesanes

**Figure 11:**
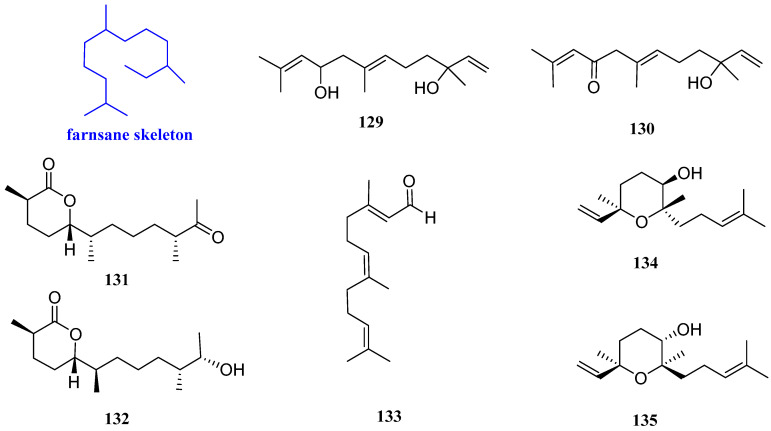
Structures of farnesane-type sesquiterpenoids **129**–**135**.

9-Hydroxynerolidol and 9-oxonerolidol (**129** and **130**, Figure 11), possessing chain-like structures, are two farnesane-type sesquiterpenoids isolated from *Chiliadenus lopadusanus*. The difference between compounds **129** and **130** is that the C-9 hydroxyl group of **129** is oxidized in **130**. In the antibacterial experimental assay, **129** exhibited antibacterial activity against *Acinetobacter baumannii* (*A. baumannii*) *and S. aureus*, with MIC values of 150 µg/mL and 75 µg/mL, respectively. Compound **130** exhibited antibacterial activity against *A. baumannii and S. aureus*, with the same MIC value of 150 µg/mL [57].

Chermesiterpenoids B and C (**131** and **132**, Figure 11) were from the marine red algal-derived fungus *Penicillium chermesinum* *EN-480*. Compound **131** showed inhibitory effects on *V. anguillarum*, *Vibrio parahaemolyticus* (*V. parahaemolyticus*), *M. luteus*, *C. gloeosporioides*, and human pathogen *E. coli* with MIC values of 0.5, 16, 64, 32, and 64 µg/mL, respectively. Compound **132** inhibited the aquatic pathogens *V. anguillarum*, *V. parahaemolyticus*, *M. luteus*, and *C. gloeosporioides* with MIC values of 1, 32, 16, and 64 µg/mL, respectively, while had no effect against *E. coli*. The positive control amphotericin B had a MIC value of 1.0 µg/mL [58].

Farnesal (**133**, Figure 11) was isolated from the *n*-hexane fraction of the crude acetone extract from the leaves of the Australian Plant *Eremophila lucida* and showed antibacterial activity against *S. aureus* ATCC 25923 and *S. aureus* ATCC 29213, with the same MIC value of 65 µg/mL (195 µM) [59].

*Rel*-(3*R*,6*R*,7*S*)-3,7,11-trimethyl-3,7-epoxy-1,10-dodecadien-6-ol and 6*α*-hydroxycyclonerolidiol (**134** and **135**, Figure 11) were isolated from the heartwood of *Dalbergia odorifrea* T. Chen. Compound **134** was effective against *C. albicans*, with an inhibition zone diameter of 10.86 mm, and compound **135** exhibited inhibition zone diameters of 9.21 mm against *C. albicans* and 11.02 mm against *S. aureus*, respectively [60].

### 2.10. Chamigranes 

**Figure 12:**
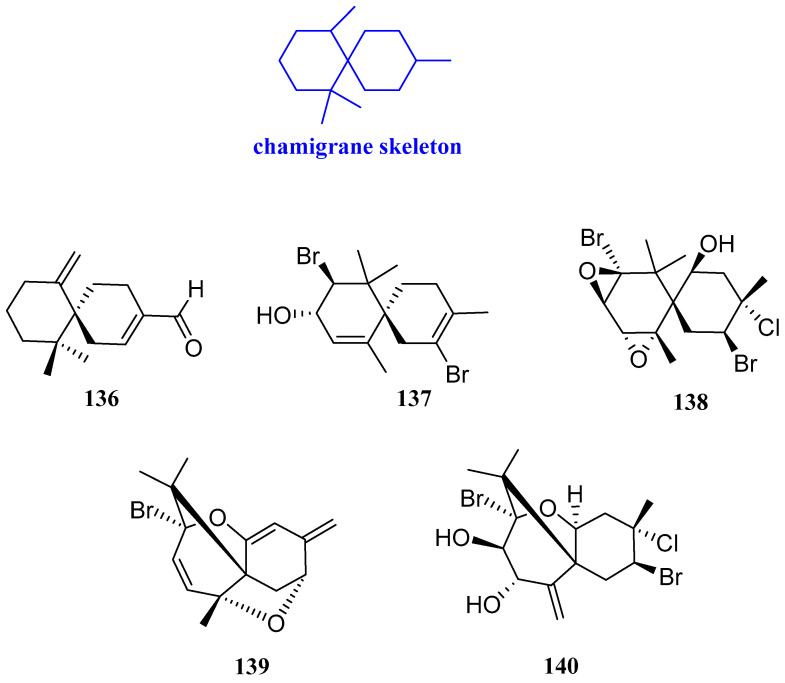
Structures of chamigrane-type sesquiterpenoid **136**–**140**.

The herb of *Leonurus japonicus* is a type of traditional Chinese medicine that contains a large number of secondary metabolites. It was often used to regulate menstruation and promote blood circulation. One sesquiterpenoid, named chamigrenal (**136**, Figure 12), was isolated and studied for its antibacterial activities by the microdilution method. The results showed that compound **136** had antibacterial activity against *E. coli*, *E. aerogenes*, *Macrococcus caseolyticus* (*M. caseolyticus*), *S. auricularis*, and *S. aureus*, and the MIC value was in the range from 25 to 200 µg/mL [56]. 2,10*β*-Dibromochamigra-2,7-dien-9*α*-ol, prepacifenol epoxide, compositacin N, and pacifenediol (**137**−**140**, Figure 12) were isolated from the red alga *Laurencia tristicha.* Compounds **137**−**140** had inhibitory activity against *S. aureus, M. gypseum,* and *T. rubrum*, with MIC values of 16−118 μg/mL [17].

### 2.11. Pseudoguaiane

**Figure 13:**
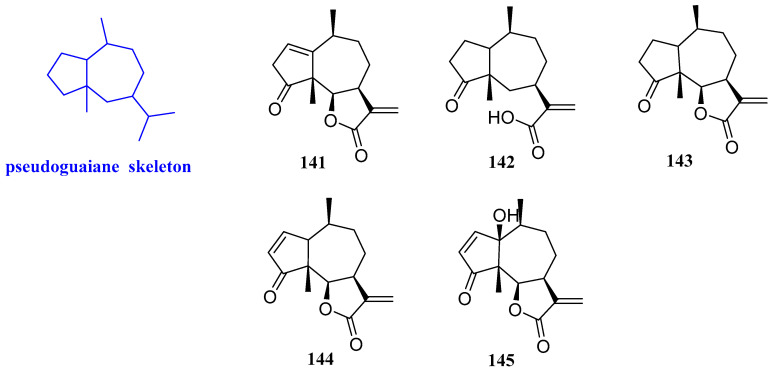
Structures of pseudoguaiane-type sesquiterpenoids **141**–**145**.

Five sesquiterpenoids were isolated from chloroform extract of *Ambrosia maritima*, including neoambrosin, damsinic acid, damsin, ambrosin, and hymenin (**141**–**145**, Figure 13). The antibacterial assays showed that these five sesquiterpenoids had certain antibacterial effects against two plant pathogenic bacteria, *Agrobacterium tumefaciens* (*A. tumefaciens*) and *E. carotovora*, with MIC values ranging from 90 to 520 mg/L. Compound **141** was the most effective against *A. tumefaciens* and *E. carotovora*, with MIC values of 150 and 90 mg/L, respectively. In addition, compound **145** caused significant activation of *E. carotovora* enzymes [61].

### 2.12. Drimanes

**Figure 14:**
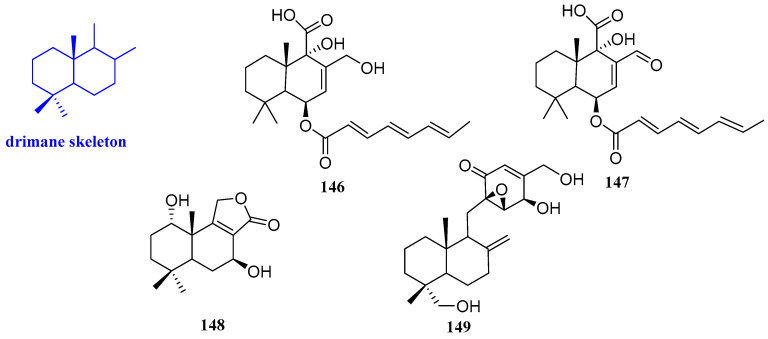
Structures of drimane-type sesquiterpenoids **146**−**149**.

Two new sesquiterpenoids named ustusoic acid A and B (**146** and **147**, Figure 14) were isolated from *Aspergillus ustus*. These compounds had a weak inhibitory effect on vancomycin-resistant *Enterococcus faecium* (*E. faecium*) ATCC 700221 and *B. subtilis* ATCC 49343. Compounds **146** and **147** had a weak effect on *B. subtilis* ATCC 49343 and vancomycin-resistant *E. faecium* ATCC 700221, with MIC values ranging from 38 to 128 µg/mL, respectively [62].

(1*S*,5*S*,7*S*,10*S*)-Dihydroxyconfertifolin (**148**, Figure 14) was obtained from *Talaromyces purpureogenu* residing inside the plant *Panax notoginseng,* which had an inhibitory effect on *E. coli* with the MIC value of 25 µM/L [63]. 13-Hydroxylmacrophorin A (**149**, Figure 14) was isolated from the endophyte *Microdiplodia* *sp.* TT-12. Compound **149** had weak activity against *R. quercivora*, whereas it showed moderate antimicrobial activity against both *P. aeruginosa* ATCC 15442 and *S. aureus* NBRC 13276. The results implied that compound **149** is an ingredient that has an antimicrobial against *R. quercivora* JCM 11526, with an inhibitory area of 12 mm in the culture of *Microdiplodia sp.* TT-12, which was isolated from the plant hosts [49].

### 2.13. Aromadendrane

**Figure 15:**
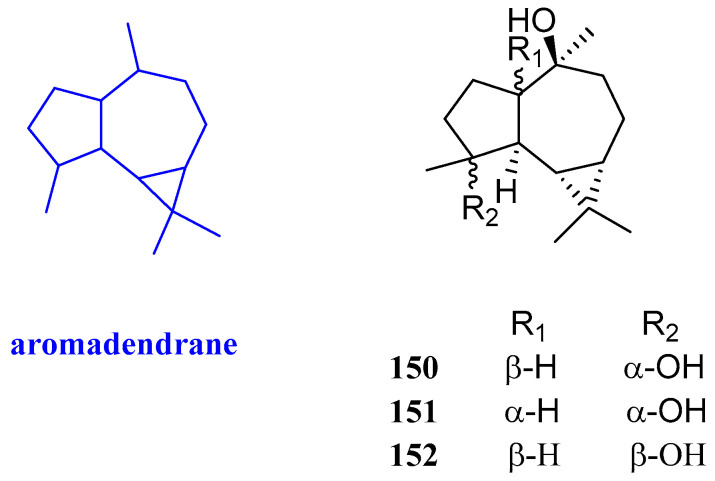
Structures of aromadendrane-type sesquiterpenoid **150**−**152**.

Aromadendrane-4*β*,10*α*-diol, aromadendrane-4*α*,10*α*-diol, and 1-epimer-aromadendrane-4*β*,10*α*-diol (**150**–**152**, Figure 15) were isolated from *Cassia* buds, the immature fruits of *Cinnamomum cassia* (Lauraceae), and their antibacterial activity was evaluated. Compound **151** showed selective inhibitory activities against *S. aureus*, with an inhibitory zone diameter of 8 mm, while it had no activity against *C. albicans* and *E. coli*. Compound **150** exhibited inhibitory effects against *C. albicans, S. aureus*, and *E. coli*, and the inhibitory zone diameters were 10, 7, and 10 mm, respectively. Compound **152** not only inhibited the proliferation of *C. albicans* but also inhibited the proliferation of *S. aureus*, with inhibitory zone diameters of 10 and 8 mm, respectively [30].

### 2.14. Cuparanes

**Figure 16:**
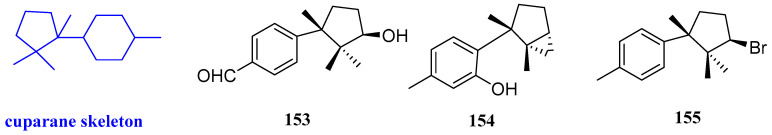
Structures of cuparane-type sesquiterpenoids **153**–**155**.

*Laurencia obtusa* lamouroux is a marine species with a variety of biological activities, including antioxidant, antibacterial, and so on. 10-Hydroxycuparaldehyde (**153**, Figure 16) from *L. obtusa* lamouroux had good inhibitory activity, with MIC values in the range of 0.08–0.15 mM, for *E. coli*, *Klebsiella pneumoniae* (*K. pneumoniae*), *P. mirabilis*, *P. aeruginosa*, *E. faecalis*, and *S. aureus* [36].

Debromolaurinterol and *α*-bromocuparane (**154** and **155**, Figure 16) was isolated from Bornean *Laurencia snapeyi*. It was found that compounds **154** and **155** had good antibacterial activity against *S. typhi*, with a MIC/MBC ratio of 2.79 and 2.72, indicating a bactericidal antibiosis [64].

### 2.15. Daucanes

**Figure 17:**
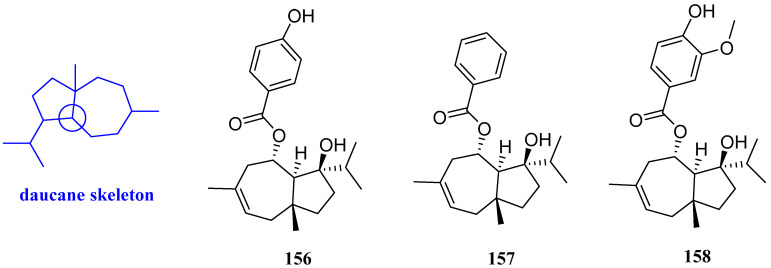
Structures of daucane-type sesquiterpenoids **156**–**158**.

Jaeschkeanadiol *p*-hydroxybenzoate (ferutinin), jaeschkeanadiol benzoate (teferidin), and jaeschkeanadiol vanillate (teferin) (**156**–**158**, Figure 17) were obtained from the root of *Ferula hermonis*. Compounds **156**–**158** possessed antibacterial effects on *MRSA, B. subtilis, Mycobacterium tuberculosis* (*M. tuberculosis*)*,* and *M. bovis*, with MIC values ranging from 0.39 to 8 µg/mL. In this study, positive controls including tetracycline, isoniazid, ciprofloxacin, and chloramphenicol were used; however, no comparison was addressed between the potency of antibiotics and those of compounds **156**–**158** [65].

### 2.16. Illudalanes

**Figure 18:**
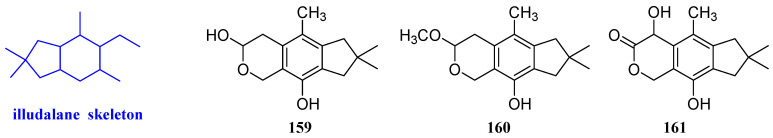
Structures of illudalane-type sesquiterpenoids **159**–**161**.

The antibacterial activity and cytotoxicity of incarnatin A, incarnatin B, and incarnolactone C (**159**–**161**, Figure 18), which were isolated from the mushroom Gloeostereum incarnatum BCC41461, were tested. Compound **161** exhibited anti-B. cereus activity, with a MIC value of 25 μg/mL, while the MIC values of compounds **159** and **160** were both more than 25 μg/mL [66].

### 2.17. Oplopananes

**Figure 19:**
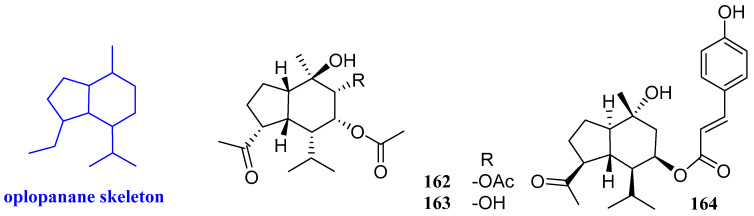
Structures of oplopanane-type sesquiterpenoids **162**–**164**.

Two new sesquiterpenoids were isolated from the ethyl acetate extract of *Chimonanthus praecox* link, named chimonols A and B (**162** and **163**, Figure 19). The antimicrobial activities of these two compounds were evaluated and the minimum inhibitory concentrations (MICs) were determined by the broth microdilution method in 96-well culture plates. The results suggested that compounds **162** and **163** had a weak antibacterial effect on *S. aureus* ATCC 6538 and *S. aureus* ATCC 25923, and the MIC values were 158.2–223.8 µg/mL. Compounds **162** and **163** were inactive against *M. tuberculosis*, with MIC values being greater than 250 μg/mL [67].

8-*β*-*p*-Coumaroyl-oplopanone (**164**, Figure 19) was isolated from the ethanol extract of the whole herbs of *Pilea cavaleriei*. An antibacterial experiment revealed that compound **164** had anti-tuberculosis activity, and the MIC value was 16 µg/mL [68].

### 2.18. Picrotoxanes

**Figure 20:**
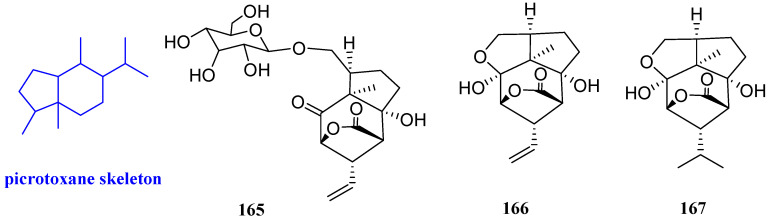
Structures of picrotoxane-type sesquiterpenoids **165**–**167**.

Three sesquiterpenoids including ramifloside, sapidolide A, and picrotoximaesin (**165**–**167**, Figure 20) were isolated from the fruit of *Bacurea ramiflora.* These three compounds exhibited an inhibitory effect on *C. gloeosporioides*, and the MIC values were 12.5, 12.5, and 50 μg/mL respectively [69].

### 2.19. Rhodolauranes

**Figure 21:**
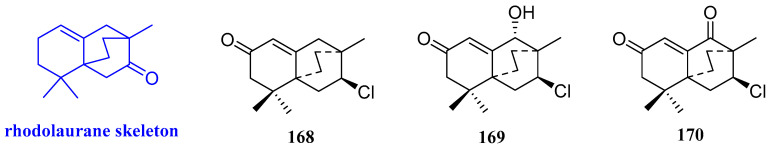
Structures of rhodolaurane-type sesquiterpenoids **168**–**170**.

Rhodolaurenones A–C (**168**–**170**, Figure 21) were isolated from Bornean *Laurencia majuscula* (Harvey) Lucas. Compounds **169** and **170** displayed bactericidal activity against *E. coli, S. typhi,* and *V. cholera*, with a MIC value of 100 μg/mL and an MBC value of 250 μg/mL. Compound **168** had a MIC value of 250 μg/mL and an MBC value of 1000 μg/mL, respectively, against *E. coli* [70].

### 2.20. Others

**Figure 22:**
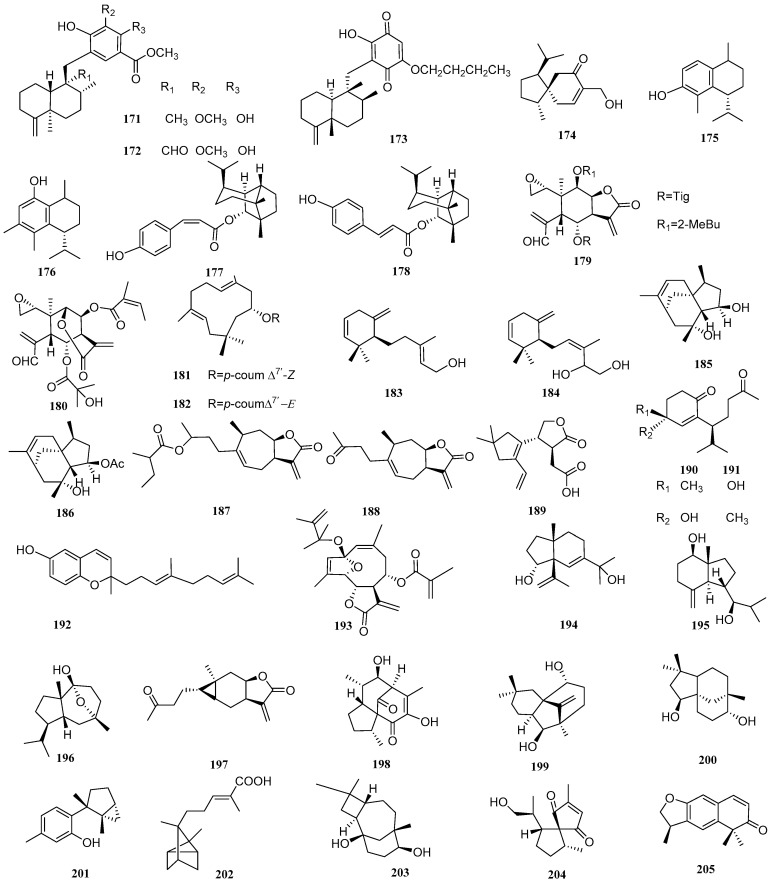
Structures of sesquiterpenoids **171**–**205** of other types.

Three sesquiterpenoids were isolated from a Vietnamese marine sponge of *Spongia* *sp.* and named as langconols A and C and langcoquinone C (**171***–***173**, Figure 22), respectively. The antibacterial assays of these isolates suggested that **171** and **172** possessed significant antibacterial activities against *B. subtilis*, with MIC values of 12.5 and 25 µM, and **173** also had good inhibitory effects against *B. subtilis* and *S. aureus*, with MIC values of 6.25 and 12.5 µM, respectively [71]. Compound **174** (Figure 22), named 4-*epi*-15-hydroxyacorenone, from Chinese agarwood, could inhibit the proliferation of *S. aureus* and *R. solanacearum*, with inhibitory zones of 12.35 and 16.9 mm [48]. Two sesquiterpenoids, dysoxyphenol and 7*R*,10*S*-2-hydroxycalamenene (**175** and **176**, Figure 22), were isolated from the acetone extract of *Dysoxylum densiflorum* seeds. Both compounds had significant antibacterial properties against *B. subtilis* (MIC = 28 μM) which were better than those of the positive control amoxicillin (MIC = 34 µM). Compounds **175** and **176** were also evaluated for their antifungal properties against two wood-rotting fungi (brown rot, *F. palustris*; white rot, *T. versicolor*) using a zone inhibition assay at two concentrations (0.46 and 4.58 mM). Compound **175** showed the same antifungal effect to both fungi at both concentrations. Compound **176** was able to inhibit the growth of white-rot fungi but not brown-rot fungi at the concentration of 0.46 mM and inhibited both fungi at a higher concentration (4.58 mM) [72].

(1*R*,2*S*,5*S*,6*S*,7*S*,10*R*)-1-*O*-[(*Z*)-*p*-Coumaroyl]-copaborneol and (1*R*,2*S*,5*S*,6*S*,7*S*,10*R*)-1-*O*-[(*E*)-*p*-coumaroyl]-copaborneol (**177** and **178**, Figure 22) were isolated from *Pilea cavaleriei*. The antibacterial activities of compounds **177** and **178** were tested, which indicated that these two compounds had moderate antimycobacterial activity against *M. tuberculosis* H37Rv, with MIC values of 4.84 and 9.83 µg/mL, respectively [73].

New sesquiterpenoid lactones, zinaflorin VI and the *δ*-elemenolide juniperin (**179** and **180**, Figure 22), were isolated from *Zinnia peruviana* L. The MICs of 179 on *B. subtilis* and *S. aureus* were 32 and 64 µg/mL, respectively, and the MICs were 4 and 8 µg/mL for compound **180** while the *α*-Glucosidase inhibition was not active [74].

(1*E*,5*E*,8*R*)-8-*O*-[(*Z*)-*p*-Coumaroyl]humula-1(10),4(5)-dien-8-ol and (1*E*,5*E*,8*R*)-8-*O*-[(*E*)-*p*-coumaroyl]humula-1(10),4(5)-dien-8-ol (**181** and **182**, Figure 22) were isolated from *Pilea cavaleriei*. Compounds **181** and **182** showed moderate antimycobacterial activity against *M. tuberculosis* H37Rv, with MIC values of 3.75 and 7.28 µg/mL, respectively [73]. Genus *Laurencia* is often studied by researchers, and it has large number of non-secondary metabolites. Two sesquiterpenoids were isolated from Bornean *Laurencia snapeyi*, including snakeol and snakediol (**183** and **184**, Figure 22). Researchers tested the antibacterial activity of the two compounds by the microdilution method. The result revealed that compounds **183** and **184** showed strong antibacterial activity against *E. coli*, with MIC/MBC ratios of 3.02 and 2.76, respectively [64].

Two compounds named penicibilaenes A and B (**185** and **186**, Figure 22) were obtained from the marine isolate of *Penicillium bilaiae* MA-267. Both compounds have selective inhibitory effects on *C. gloeosporioides*, with MIC values of 1.0 and 0.125 μg/mL, respectively [75].

*Carpesium macrocephalum* has the characteristic of killing fungi. Two sesquiterpenoids, named 4-(2-methybutyryl)-4*H*-tomentosin and tomentosin (**187** and **188**, Figure 22), were extracted and isolated from *C. macrocephalum*. Compounds **187** and **188** inhibited the yeast-to-hyphae morphogenetic transition of *C. albicans*, with IC_50_ values of 105.1 and 31.6 μg/mL [33].

A new sesquiterpenoid, named leptosphin B (**189**, Figure 22), was isolated from the solid fermentation cultures of an endophytic fungus, *Leptosphaeria* sp. XL026, isolated from the leaves of *Panax notoginseng*. Compound **189** showed antibacterial activity against *B. cereus*, with MIC values of 12.5 μg/mL [43]. Chimonols C and D (**190** and **191**, Figure 22) were extracted from the ethyl acetate extract of *Chimonanthus praecox* Link. The broth microdilution method was used to test the antibacterial ability. Compound **190** showed activity against *S. aureus* (ATCC 43300 and ATCC 25923) and *C. glabrata* (ATCC 2001), with MIC values from 128 to 162 µg/mL. The MIC values of compound **190** against *S. aureus* (ATCC 25923) and *C. glabrata* (ATCC 2001) were 183−254 µg/mL. However, both compounds **190** and **191** were inactive against *M. tuberculosis*, with MIC values over 250 µg/mL [67].

Researchers tested the antibacterial activity and cytotoxicity of (*E*)-dictyochromenol (**192**, Figure 22), which was isolated from the brown alga *Dictyopteris undulate* Holmes. The result found was that compound **192** displayed anti-*B. cereus* activity, with a MIC value of 1.56 μg/mL [66]. An antibacterial sesquiterpenoid compound from *Elephantopus tomentosus*, named tomenphantopin H (**193**, Figure 22), was isolated, possessing an inhibitory effect on *S. aureus*, with a diameter of the inhibition zone of 14.2 mm, while the diameter of the inhibition zone of the positive control, Kanamycin sulfate, was 32.6 mm [76].

Two compounds, cinnamosim A and 1*β*,7-dihydroxyl opposite-4(15)-ene (**194** and **195**, Figure 22), were isolated from *Cassia* buds, the immature fruits of *Cinnamomum cassia* (Lauraceae). The antibacterial activities of compounds **194** and **195** were evaluated. The result was that compound **194** and **195** selectively inhibited the proliferation of *C. albicans*, with inhibitory zone diameters of 11 and 8 mm, respectively, at the concentration of 300 μg/disk. Compound **195** could also inhibit the proliferation of *S. aureus*, with inhibitory zone diameters of 7 mm at the same concentration [30]. 10-Hydroxy-7,10-epoxysalvialane (**196**, Figure 22) with antibacterial effects was obtained from *Alisma orientale* and could inhibit *S. aureus*, with a MIC value of 100 mg/mL [31].

Carabrone (**197**, Figure 22) was extracted and isolated from *Carpesium macrocephalum*. The antibacterial experiment showed that compound **197** had inhibitory activity against *C. albicansi* and inhibited the yeast-to-hyphae morphogenetic transition through microscopic observation, with an IC_50_ value of 100.1 μg/mL [33]. Rhodocorane L (**198**, Figure 22), isolated from the fermentation broth of the basidiomycete *Rhodotus palmatus,* had medium antifungal ability on *N. coryli* and *R. glutinis*, with the same MIC value of 66.7 μg/mL [77]. Antroalbocin A (**199**, Figure 22) was isolated from *Antrodiella albocinnamoea* and could inhibit *S. aureus*, with a MIC value of 169 µM [78].

Clovane-2*β*,9*α*-diol (**200**, Figure 22) was isolated from *Eugenia jambolana* seeds. It was found that compound **200** had inhibitory activity against *S. aureus*, with inhibitory zone diameters of 10 mm at the concentration 100 μg/disk. [28]. Debromolaurinterol (**201**, Figure 22) was isolated from the red algae *Laurencia snackeyi*. The antibacterial activity of compound **201** was tested by the microdilution method. The result found was that compound **201** showed strong antibacterial activity against *S. typhi*, with a MIC/MBC ratio of 2.79 [64].

(+)-(*E*)-*α*-Santalen-12-oic-acid (**202**, Figure 22) was isolated from methanol extract of the stem and leaf of *Clausena lansium*. Compound **202** had weak antibacterial activity against *B. cereus*, with an IC_50_ value of 74.6 µM [79]. Caryolane-1,9*β*-diol (**203**, Figure 22) was isolated from Cassia buds, the immature fruits of *Cinnamomum cassia* (Lauraceae). Compound **203** had inhibitory effects on *C. albicans*, *S. aureus,* and *E. coli*, with inhibitory zone diameters of 10, 8.5, and 7 mm, respectively, at the concentration of 300 μg/disk [30]. Rhodocorane K (**204**, Figure 22) was found to have medium antifungal ability against *N. coryli* DSM 6981 and *R. glutinis* DSM 10134, with the same MIC values of 66.7 μg/mL [77]. Variabilone (**205**, Figure 22) was found in the endophytic fungus *Paraconiothyrium variabr* and could well inhibit *B. subtilis*, with an IC_50_ value of 2.13 µg/mL [80].

## 3. Mechanisms of Antimicrobial Action by Sesquiterpenoids

The mechanisms of antibiotics against bacteria mainly include affecting cell wall synthesis (*β*-lactams) and disrupting bacterial membranes, interacting with ribosomal subunits (Tetracycline, Chloramphenicol, Aminoglycosides, etc), disrupting nucleic acid action (Rifampicin, Fluoroquinolones), and interfering with metabolic pathways (Folic acid analogs, sulfonamides) [81]. The corresponding mechanism of antibacterial resistance ranges from accelerating antibiotic efflux through bacterial efflux pumps; alteration of the bacterial porins’ structure, which decreases bacterial permeability to antibiotic influx; and destruction of antibacterial agents by hydrolytic enzymes to alteration of binding sites for antibiotics [82].

The mechanism of sesquiterpenoids against bacteria has not been clearly reported, but it is believed that the microbial cell membranes play an important role. Bacterial subpopulations which are characterized by low metabolism could reduce absorption of antibiotics, especially for the active molecules on the cell wall such as beta-lactams and glycopeptides, making it difficult to treat infections. The mechanism by which the sesquiterpenoids can inhibit the microorganisms involves different modes of action; one which researchers basically agree with is that sesquiterpenoids can destabilize microbial cell membranes. Because the bacterial cell wall is highly lipophilic, it means that a certain lipophilicity is necessary for antibiotics to function [83]. The hydrophobicity of some sesquiterpenoids disturbs the cytoplasmic membrane or compounds in it, such as some classes of proteins, increasing the ionic permeability and causing cytoplasmic extravasation and, as consequence, cellular lysis, as well as interfering with the activity of the respiratory current and energy production. Terpenes isolated from essential oils, such as thymol and carvacrol, may act as permeabilizers of the cell membrane, increasing the entry of antibiotics [54]. Thus, *α*-bisabolol is possibly responsible for the antibacterial and synergic action when associated with antibiotics. Lipophilic sesquiterpenoids can destroy the membrane and cause ion leakage in the membrane. The results showed that the action mode of *β*-caryophyllene is to damage the cell membrane and produce non-selective pores, causing the leakage of substances in the cells, and finally causing cell death [84]. Farnesal and farnesol have previously been reported to have antimicrobial activity. Farnesol exerts antibacterial activity by disrupting the cell membrane, and it was also found that it can destroy biofilms of Gram-positive bacteria by reducing biomass [59]. Although the mechanisms responsible for the antibacterial activity of farnesal have not yet been reported, it appears reasonable to hypothesize that farnesal could act in the same way as farnesol, possibly by its hydrophobic nature facilitating insertion into the bacterial phospholipid bilayer membrane and consequent structural disruption. Interestingly, bacteria are able to decrease the concentration of antibiotics in their own cells through the overexpression of efflux pumps. In a mechanistic study, Fazly Bazzaz et al. showed that galbanic acid modulated the resistance in clinical drug-resistant isolates of *S. aureus* via the inhibition of the efflux pump [85].

The main mechanisms of antifungal effects concern interfering substance transport, yeast-to-hypha transition, host immunity, redox, and others [86]. For instance, polygodial is a sesquiterpenoid dialdehyde that can inhibit fungi. M. V. Castelli et al. carried out experiments using mammalian mitochondrial preparations. The results support the claim that that polygodial mainly plays a role in inhibiting ATP synthesis because the ATP synthesis of phosphorylating submitochondrial Mg^+^-ATP particles is inhibited at the concentration of polymers similar to the MIC values reported by several yeasts and filamentous fungi [87,88].

Natural products with good inhibitory activity against both bacteria and fungi have broad application prospects in the future development of antifungal drugs. Therefore, it is necessary to clear the mechanisms of action and find antimicrobial targets while exploring new antimicrobial agents.

## 4. Structure-Activity Relationship

In this review, the structure-activity relationship of compounds with antibacterial activities was analyzed. The compounds with eremophilane, xanthane, lindenane, farnesane, guaiane, penicibilaene, germacrene, daucane, carotene, and illudalane-type skeleton showed relatively strong antibacterial activity (MIC values were lower than 50 μg/mL). Among these types, differences in substituents, different substitution sites, and configuration lead to various degrees of bacteriostatic effect.

Compound **21** had good antibacterial activity against *T. rubrum*, while compound **22** showed no activity. Apart from C6-OH, compounds **21** and **22** both had a bisabolane skeleton, which may prove the importance of C6-OH in the inhibition of this kind of fungi. Compounds **28**–**34** all have a similar guaiane skeleton. Comparing the antibacterial activity in pairs, compounds **31**, **33**, and **34** with C4-OH had strong activity for a variety of pathogenic bacteria, while other compounds without this group had slightly lower antibacterial activity. It seems that C4-OH increased antibacterial activity. In the antibacterial experiment, **71** and **72** are both active against *S. aureus*, with **71** being more potent than **72**. The difference in structures is that the C-9 hydroxyl of **71** was oxidized in **72**, which may influence the activity. For farnesanes-type sesquiterpenoids, compound **73** was more active against aquatic and human pathogens than compound **74**, but less active against the plant pathogenic fungus, which may be due to the different oxidation degrees of the compounds under C-11. For pseudoguaianes-type sesquiterpenoids, the inhibitory effects of **141** on *A. tumefaciens* and *E. carotovora* were stronger than those of **142**. The structures of **142** did not have double bonds at C2 and C3, comparing it with 141, which suggested that the double bonds on C-2 and C-3 may increase the antibacterial activity.

## 5. Discussion

**Figure 23:**
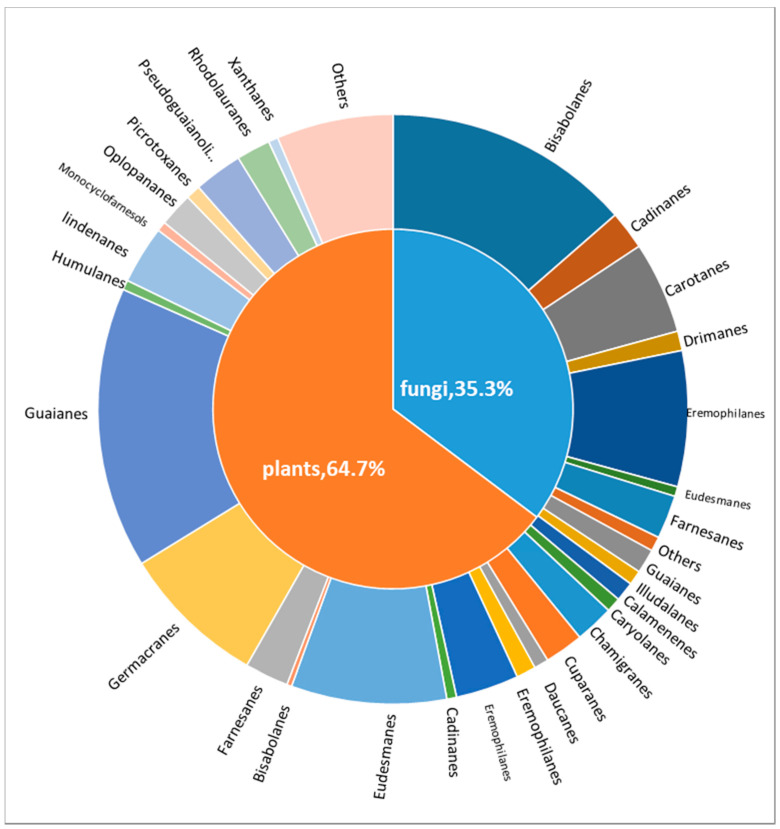
Skeletal types of antimicrobial compounds of plant and fungal origin.

This review shows that a variety of antibacterial sesquiterpenoids were isolated from plants and fungi (64.70% and 35.30%, respectively) (Figure 23). Among them, sesquiterpenoids obtained from plants were mainly distributed in guaiane, eudesmane, germacrane, eremophilane, lindenane, and pseudoguaianolide-type skeletons, with numbers of 58, 32, 30, 19, 13, 12, and 10, respectively. Sesquiterpenoids obtained from fungi were mainly distributed in bisabolane, eremophilane, carotane, farnesane, and cadinane-type skeletons, with numbers of 51, 28, 19, 9, and 8, respectively.

## 6. Conclusions

A total of 205 sesquiterpenoids with antibacterial and antifungal activity, which were found and tested from 2012 to 2022, were mainly included in 19 carbon skeleton types, and the number of guaiane sesquiterpenoids was the largest. The names, sources, and chemical structures of 205 sesquiterpenoids are listed in this review. The structure–activity relationship of active compounds is also discussed. According to the data above, we can derive some potential molecules with good antibacterial and antifungal activity. Compound 100 is considered as a potential antimicrobial compound against *E. coli*, with a MIC of 0.5 µg/mL. Compounds **114** and **134** were most potent against *B. licheniformis*, with MIC values of 3.1 and 2.3 µg/mL, respectively. Furthermore, compounds **114** and **31** also exhibited an effect on *C. albicans*, with MICs of 0.26 and 0.21 µg/mL, respectively. Compound **122** showed a striking inhibition of *F. oxysporum*. F. sp. *Cucumebrium* and *B. sorokiniana*, with the same MIC value of 1 µg/mL. As for *B. subtilis*, compound 180 showed a strong activity (MIC = 4 µg/mL). Compound **192** had a strong effect on *B. cereus* (MIC = 1.56 µg/mL). The above conclusions were drawn by reviewing large number of sesquiterpenoids and comparing the antimicrobial activities of different structures. The structure-activity relationship plays a key role in modern chemical synthesis and will help people synthesize more effective sesquiterpenoids and use safe natural compounds as antibacterial agents in overcoming bacteria, fungi, and the challenge of drug resistance. These sesquiterpenoids may have the most potential as new natural antibacterial compounds. It is hoped that this review will provide support for the discovery of active drug lead molecules.

## Supplementary Materials

The following supporting information can be downloaded at: https://www.mdpi.com/article/10.3390/biom12091271/s1, The Mol. files of sesquiterpenoids with antibacterial and antifungal activity; Table S1: Abbreviations of bacteria and fungi; Table S2: Compounds with antibacterial and antifungal effects. Table S3: Compounds with antibacterial and antifungal effects.
