# Supplementary material for: Antibacterial and Antifungal Sesquiterpenoids: Chemistry, Resource, and Activity
Source: Biomolecules. 2022 Sep 9;12(9):1271. doi: 10.3390/biom12091271 (PMC9496053; doi:10.3390/biom12091271)
Supplement: Supplementary file 1 [file biomolecules-12-01271-s001.zip › biomolecules-1887774-Table S3.docx]

**Table S3.** Compounds with antibacterial and antifungal effects.

| **No.** | **Compounds** | **Source** | **Microorganism** | **Activities** | **Ref.** |
| --- | --- | --- | --- | --- | --- |
| 1 | Laurecomposin A | The red alga Laurencia composita Yamada | *S. aureus* | MIC=26.8 μg/mL | [17] |
|  |  |  | *M. gypseum* | MIC=4 μg/mL |  |
|  |  |  | *C. albicans* SC5314 | MIC=16 μg/mL |  |
| 2 | Laurecomposin B | The red alga Laurencia composita Yamada | *S. aureus* | MIC=15.4 μg/mL | [17] |
|  |  |  | *M. gypseum* | MIC=8 μg/mL |  |
| 3 | Preintricatol | The red alga Laurencia composita Yamada | *S. aureus* | MIC=13.6 μg/mL | [17] |
|  |  |  | *M. gypseum* | MIC=8 μg/mL |  |
|  |  |  | *C. albicans* SC5314 | MIC=32 μg/mL |  |
| 4 | Helianthol B | The red alga Laurencia composita Yamada | *M. gypseum* | MIC=32 μg/mL | [17] |
|  |  |  | *S. aureus* | MIC=54 μg/mL |  |
| 5 | Gossonorol | The red alga Laurencia composita Yamada | *M. gypseum* | MIC=4 μg/mL | [17] |
|  |  |  | *S. aureus* | MIC=10.9 μg/mL |  |
| 6 | Elgonene C | A basidiomycete collected in Mount Elgon Natural Reserve | *S. aureus* DSM 346 | MIC=100 μg/mL | [18] |
|  |  |  | *M. hiemalis* DSM 2656 | MIC=100 μg/mL |  |
| 7 | Elgonene D | A basidiomycete collected in Mount Elgon Natural Reserve | *M. hiemalis* DSM 2656 | MIC=50 μg/mL | [18] |
| 8 | Elgonene G | A basidiomycete collected in Mount Elgon Natural Reserve | *M. hiemalis* DSM 2656 | MIC=100 μg/mL | [18] |
|  |  |  | *B. subtilis* DSM 10 | MIC=100 μg/mL |  |
|  |  |  | *M. luteus* DSM 1790 | MIC=100 μg/mL |  |
|  |  |  | *S. aureus* DSM 346 | MIC=100 μg/mL |  |
| 9 | Elgonene H | A basidiomycete collected in Mount Elgon Natural Reserve | *B.subtilis* DSM 10 | MIC=100 μg/mL | [18] |
|  |  |  | *M. luteus* DSM 1790 | MIC=100 μg/mL |  |
|  |  |  | *S. aureus* DSM 346 | MIC=100 μg/mL |  |
|  |  |  | *M. hiemalis* DSM 2656 | MIC=100 μg/mL |  |
| 10 | Elgonene I | A basidiomycete collected in Mount Elgon Natural Reserve | *B. subtilis* DSM 10 | MIC=75 μg/mL | [18] |
| 11 | Egonene J | A basidiomycete collected in Mount Elgon Natural Reserve | *B. subtilis* DSM 10 | MIC=75 μg/mL | [18] |
|  |  |  | *M. hiemalis* DSM 2656 | MIC=50 μg/mL |  |
| 12 | Elgonene K | A basidiomycete collected in Mount Elgon Natural Reserve | *M. hiemalis* DSM 2656 | MIC=50 μg/mL | [18] |
| 13 | Elgonene L | A basidiomycete collected in Mount Elgon Natural Reserve | *M. hiemalis* DSM 2656 | MIC=25 μg/mL | [18] |
|  |  |  | *M. luteus* DSM 1790 | MIC=100 μg/mL |  |
|  |  |  | *S. aureus* DSM 346 | MIC=100 μg/mL |  |
|  |  |  | *B. subtilis* DSM 10 | MIC=100 μg/mL |  |
| 14 | (7*S*,10*S*)-7,10-Epoxysydonic acid | *Aspergillus* sp. xy02 | *S. aureus* ATCC 25923 | IC_50_=32.2 µM | [19] |
| 15 | (7*R*,11*S*)-7,12-Epoxysydonic acid | *Aspergillus* sp. xy02 | *S. aureus* ATCC 25923 | IC_50_=36.0 µM | [19] |
| 16 | 7-Deoxy-7,14-didehydro-12-hydroxysydonic acid | *Aspergillus* sp. xy02 | *S. aureus* ATCC 25923 | IC_50_=41.9 µM | [19] |
| 17 | (*E*)-7- Deoxy-7, 8-didehydro-12-hydroxysydonic acid | *Aspergillus* sp. xy02 | *S. aureus* ATCC 25923 | IC_50_=31.5 µM | [19] |
| 18 | Engyodontiumone I | *Aspergillus* sp. xy02 | *S. aureus* ATCC 25923 | IC_50_=33.4 µM | [19] |
| 19 | (+)-Hydroxysydonic acid | *Aspergillus* sp. xy02 | *S. aureus* ATCC 25923 | IC_50_=34.0 µM | [19] |
| 20 | (−)-(7*S*)-10-Hydroxysydonic acid | *Aspergillus* sp. xy02 | *S. aureus* ATCC 25923 | IC_50_=36.3 µM | [19] |
| 21 | (+)-Phomoterpene A | *Phomopsis prunorum* (F4-3) | *P. syringae* pv. *Lachrymans* | MIC=15.6 µg/mL | [20] |
|  |  |  | *X. citri* pv*.* *phaseoli var. fuscans* | MIC=62.4 µg/mL |  |
| 22 | (−)-Phomoterpene A | *Phomopsis prunorum* (F4-3) | *P. syringae* pv. *Lachrymans* | MIC=31.2 µg/mL | [20] |
|  |  |  | *X. citri* pv. *phaseoli var. fuscans* | MIC=31.2 µg/mL |  |
| 23 | *α*-Bisabolol | *Vanillosmopsis arborea* Barker | *Candida* 40006 | IC_50_=8.92 µg/mL | [21] |
|  |  |  | *Candida* 40095 | IC_50_=18.15 µg/mL |  |
|  |  |  | *Candida* 40042 | IC_50_=18.26 µg/mL |  |
| 24 | (*E*)-(2*S*,3*S*,6*R*)-Atlantone-2,3-diol | *Cedrus deodara* Loud | *A. sydowii* | MIC=6400 µg/mL | [22] |
|  |  |  | *A. parasiticus* | MIC=3200 µg/mL |  |
| 25 | (*E*)-(2*S*,3*S*,6*S*)-Atlantone-2,3,6-triol | *Cedrus deodara* Loud | *T. rubrum* | MIC=125 µg/mL | [22] |
| 26 | Atlantolone | *Cedrus deodara* Loud | *A. niger, A. sydowii, A. parasiticus, A. ochraceous, A. flavus* | MICs=200–800 µg/mL | [22] |
| 27 | (*E*)-*α*-Atlantone | *Cedrus deodara* Loud | *A. niger, A. sydowii, A. parasiticus, A. ochraceous, A. flavus* | MICs=200–800 µg/mL | [22] |
| 28 | Artemivestinolide G | *Artemisia vestita* | *B. cinerea* | MIC=256 mg/L | [23] |
| 29 | Dehydrocostus lactone | *Artemisia vestita* | *F. oxysporum* | MIC=256 mg/L | [23] |
| 30 | Dihydroestafiatone | *Artemisia vestita* | *B. cinerea* | MIC=256 mg/L | [23] |
| 31 | 4*α*,9*α*,10*α*-Trihydroxyguaia-11(13)en-12,6*α*-olide | *Aerial Parts of Anvillea garcinii* | *C. albicans* | MIC=0.21 µg/mL | [24] |
|  |  |  | *C. parapsilosis* | MIC=0.25 µg/mL |  |
|  |  |  | *S. aureus* | MIC=2.3 µg/mL |  |
|  |  |  | *B. licheniformis* | MIC=2.3 µg/mL |  |
|  |  |  | *E. fergusonii* | MIC=5.7 µg/mL |  |
| 32 | 8-*O*-[3’-Hydroxy-2’-methylpropionate] | The aerial parts of *Centaurea rhizantha* | *S. aureus* | MIC/MBC=500 µg/mL | [25] |
| 33 | 6*α*-[4’,5’-Dihydroxytigloyloxy]-inuviscolide | *Schkuhria pinnata* (Lam.) Kuntze ex thell | *E. coli* | IC_50_=125 µg/mL | [26] |
|  |  |  | *P. aeruginosa* | IC_50_=46.88 µg/mL |  |
|  |  |  | *E. faecalis* | IC_50_=125 µg/mL |  |
|  |  |  | *S. aureus* | IC_50_=62.5 µg/mL |  |
| 34 | 6*α*-[4’,5’-Dihydroxytigloyloxy]-isoinuviscolide | *Schkuhria pinnata* (Lam.) Kuntze ex thell | *E. coli* | IC_50_=125 µg/mL | [26] |
|  |  |  | *P. aeruginosa* | IC_50_=46.88 µg/mL |  |
|  |  |  | *E. faecalis* | IC_50_=125 µg/mL |  |
|  |  |  | *S. aureus* | IC_50_=62.5 µg/mL |  |
| 35 | 6-Acetoxy-10-*β*-hydroguaiantrienolide | *Cotula cinerea* | *E. faecalis* 91804 | MIC=150 µg/mL | [27] |
|  |  |  | *E. faecalis* 91823 | MIC=300 µg/mL |  |
|  |  |  | *E. faecalis* 165 |  |  |
|  |  |  | *E. faecalis* 91705 |  |  |
|  |  |  | *E. faecalis* ATCC 29212 |  |  |
| 36 | 6-Acetoxy-1*α*-hydroguaiantrienolide | *Cotula cinerea* | *E. faecalis* 91804 | MIC=150 µg/mL | [27] |
|  |  |  | *E. faecalis* 91823 | MIC=300 µg/mL |  |
|  |  |  | *E. faecalis* 165 | MIC=300 µg/mL |  |
|  |  |  | *E. faecalis* 91705 | MIC=300 µg/mL |  |
|  |  |  | *E. faecalis* ATCC 29212 | MIC=300 µg/mL |  |
| 37 | Sootepdienone | The seeds of *Eugenia jambolana* fruit | *S. aureus* | IZ= 9 mm | [28] |
| 38 | Jambolanins E | The seeds of *Eugenia jambolana* fruit | *S. aureus* | IZ= 10 mm | [28] |
| 39 | Jambolanins F | The seeds of *Eugenia jambolana* fruit | *S. aureus* | IZ= 10 mm | [28] |
| 40 | Guaianediol | The seeds of *Eugenia jambolana* fruit | *S. aureus* | IZ= 9 mm | [28] |
| 41 | Wenyujinin Q | *Curcuma wenyujin* | *A. brassicicola* | MIC=50 µg/mL | [29] |
|  |  |  | *P. parasitica var. nicotianae* | MIC=100 µg/mL |  |
|  |  |  | *C. capsici* | MIC=50 µg/mL |  |
|  |  |  | *B. oryzae* | MIC=50 µg/mL |  |
|  |  |  | *D. medusaea Nitschke* | MIC=100 µg/mL |  |
|  |  |  | *C. paradoxa Moreau* | MIC=50 µg/mL |  |
|  |  |  | *E. turcicum* | MIC=25 µg/mL |  |
|  |  |  | *P. theae* | MIC=25 µg/mL |  |
|  |  |  | *A. citri* | MIC=100 µg/mL |  |
| 42 | 4*α*-10*α*-Dihydroxy-5*β*-*H*-guaja-6-ene | *Cassia buds* | *C. albicans* | IZ=9 mm | [30] |
|  |  |  | *S. aureus* | IZ=7.5 mm |  |
| 43 | 4*β*, 10*β*-Dihydroxy-1*α*H, 5*β*H-guaia-6-ene | The rhizome of *Alisma orientale* | *B. subtilis* | MIC=50 µg/mL | [31] |
| 44 | Guai-9-en-4*β*-ol | The stem of *Syringa pinnatifolia* Hemsl. var. *alashanensis* | *E. coli* | IZ=11.02 mm | [32] |
|  |  |  | *S. aureus* | IZ=13.41 mm |  |
|  |  |  | *B. coagulas* | IZ=15.34 mm |  |
|  |  |  | *P. vulgaris* | IZ=9.67 mm |  |
|  |  |  | *P. digitatum* | IZ=12.56 mm |  |
|  |  |  | *F. oxysporum* | IZ=11.64 mm |  |
|  |  |  | *A. niger* | IZ=13.20 mm |  |
| 45 | 14,15-Dinorguai-1,11-dien-9,10-dione | The stem of *Syringa pinnatifolia* Hemsl. var. *alashanensis* | *E. coli* | IZ=15.34 mm | [32] |
|  |  |  | *S. aureus* | IZ=9.45 mm |  |
|  |  |  | *B. coagulas* | IZ=12.01 mm |  |
|  |  |  | *P. vulgaris* | IZ=14.96 mm |  |
|  |  |  | *P. digitatum* | IZ=12.34 mm |  |
|  |  |  | *F. oxysporum* | IZ=15.32 mm |  |
|  |  |  | *A. niger* | IZ=11.53 mm |  |
| 46 | 4*α*,5*α*-Epoxy-10*α*,14*H*-1-*epi*-inuviscolide | Carpesium macrocephalum | *C. albicans* SC5314 | IC_50_=38 μg/mL (inhibited biofilm formation) and 106.5 (the yeast-to-hyphae morphogenetic transition) | [33] |
| 47 | *(*1*S,*2*S,*4*S,*5*S,*7*R,*10*R)-*Guaiane*-*2,10,11,12*-*tetraol | Endophytic Fungus *Xylaria* sp. YM 311643 of Azadirachta indica A. Juss. | *C. albicans*, *P. oryzae*, *A. niger* and *H. compactum* | MICs=128–512 μg/mL | [34] |
| 48 | (1*S,*2*S,*4*R,*5*R,*7*R,*10*R)-*Guaiane-2,4,10,11,12-pentaol | Endophytic Fungus *Xylaria* sp. YM 311643 of Azadirachta indica A. Juss. | *C. albicans*, *P. oryzae* and *H. compactum* | MICs=128–256 μg/mL | [34] |
| 49 | (1*S,*4*R,*5*S,*7*R,*10*R*)-Guaiane-4,5,10,11,12-pentaol | Endophytic Fungus *Xylaria* sp. YM 311643 of Azadirachta indica A. Juss. | *C. albicans*, *P. oryzae*, *A. niger* and *H. compactum* | MICs=64–256 μg/mL | [34] |
| 50 | (1*R,*4*S,*5*R,*7*R,*10*R*)-Guaiane-1,5,10,11,12-pentaol | Endophytic Fungus *Xylaria* sp. YM 311643 of Azadirachta indica A. Juss. | *C. albicans*, *P. oryzae*, *A. niger* and *H. compactum* | MICs=32–256 μg/mL | [34] |
| 51 | (1*R,*4*R,*5*R,*7*R,*10*R*)-11-Methoxyguaiane-4,10,12-triol | Endophytic Fungus *Xylaria* sp. YM 311643 of Azadirachta indica A. Juss. | *C. albicans*, *P. oryzae*, *A. niger* and *H. compactum* | MICs=32–256 μg/mL | [34] |
| 52 | Sutchuenin J | The stems and roots of *Thuja sutchuenensis.* | *B. cereus* ATCC 10876 | MIC=25 µg/mL | [35] |
|  |  |  | *S. epidermidis* ATCC 12228 | MIC=25 µg/mL |  |
| 53 | Artemivestinolide D | *Artemisia vestita* | *Pyricularia oryzae* | MIC=128 mg/L | [23] |
|  |  |  | *B. cinerea* | MIC=256mg/L |  |
| 54 | Artemivestinolide E | *Artemisia vestita* | *B. cinerea* | MIC=256mg/L | [23] |
| 55 | Artemivestinolide F | *Artemisia vestita* | *F. oxysporum* | MIC=256mg/L | [23] |
| 56 | Eudesma 4(15), 11-diene-5,7-diol | *Laurencia obtusa* | *C. albicans* | MIC=8.27 µM | [36] |
|  |  |  | *C. tropicalis* | MIC=10.13 µM |  |
| 57 | Eutyscoparin G | *Eutypella scoparia* SCBG-8 | *S. aureus* | MIC=6.3 µg/mL | [37] |
|  |  |  | Methicillin-resistant *S. aureus* | MIC=6.3 µg/mL |  |
| 58 | 1*R*,8*S*-Dihydroxy-11*R*,13-dihydrobalchanin | *Artemisia Sieberi* | *B. subtilis*, *S. aureus*, *E. coli*, *F. solani*, *P. aeruginosa*, *C. tropicalis* and *F. solani* | IZ=6–8 mm | [38] |
| 59 | (4*αβ*,7*β*,8*αβ*)-3,4,4*α*,5,6,7,8,8*α*-Octahydro-7-[1-(hydroxymethyl)ethenyl]-4*α*-methylnaphthalene-1-car boxaldehyde | Chinese agarwood | *S.aureus* | IZ=9.12 mm | [39] |
|  |  |  | *R. solanacearum* | IZ=8.98 mm |  |
| 60 | 12,15-Dioxo-*α*-selinen | Chinese agarwood | *S.aureus* | IZ=20.02 mm | [39] |
|  |  |  | *R. solanacearum* | IZ=11.02 mm |  |
| 61 | *(*5*S,*7*S,*9*S,*10*S)-*(+)-9-Hydroxy-selina-3,11-dien-12-al | Chinese agarwood | *S.aureus* | IZ=12.90 mm | [39] |
|  |  |  | *R. solanacearum* | IZ=18.20 mm |  |
| 62 | (5*S,*7*S,*9*S*,10*S*)-(+)-9-Hydroxy-eudesma-3,11(13)-dien-12-methyl ester | Chinese agarwood | *S.aureus* | IZ=14.20 mm | [39] |
|  |  |  | *R. solanacearum* | IZ=10.15 mm |  |
| 63 | (7*S,*8*R*,10*S*)-(+)-8,12-Dihydroxy-selina-4,11-dien-14-al | Chinese agarwood | *S.aureus* | IZ=8.10 mm | [39] |
| 64 | 4(15)-Eudesmene-1*β*,7,11-triol | *Cassia buds* | *C. albicans* | IZ= 9.00 mm | [30] |
| 65 | 1*β*,6*α*-Dihydroxyeudesm-4(15)-ene | *Cassia buds* | *E. coli* | IZ= 8.5 mm | [30] |
|  |  |  | *C. albicans* | IZ=11 mm |  |
|  |  |  | *S. aureus* | IZ=11 mm |  |
| 66 | Cinnamosim B | *Cassia buds* | *C. albicans* | IZ=10 mm | [30] |
|  |  |  | *S. aureus* | IZ=9 mm |  |
| 67 | 5*α*-Epoxyalantolactone | *Carpesium macrocephalum* | *C. albicans* SC5314 | IC_50_=118.4 μg/mL (inhibited the yeast-to-hyphae morphogenetic transition) | [33] |
| 68 | Telekin | *Carpesium macrocephalum* | *C. albicans* SC5314 | IC_50_=36 μg/mL (inhibited biofilm formation) | [33] |
| 69 | Ivalin | *Carpesium macrocephalum* | *C. albicans* SC5314 | IC_50_=15.40 μg/mL (inhibited biofilm formation) | [33] |
| 70 | 8-Acetoxyl-pathchouli alcohol | *The roots of Valeriana jatamansi Jones* | *S. aureus* | MIC=128 µg/mL | [40] |
|  |  |  | *P. aeruginosa* | MIC=64 µg/mL |  |
| 71 | Chlojaponol B | *Chloranthus japonicus* | *B. cinerea* | IZ=22 mm | [41] |
|  |  |  | *S. sclerotiorum* | IZ=25 mm |  |
| 72 | Cryptomeridiol | *Eugenia jambolana* | *S. aureus* | IZ=8 mm | [28] |
| 73 | 1*β*,10*β*-Epoxy-6*β*,8*α*-dihydroxyeremophila-7(11)-en-8*β*(12)-olide | *Ligularia sagitta* | *B. cereus*, *S. aureus*, *B. subtilis*, *E. coli*, and *E. carotovora* | MICs=7.25‒125 µg/mL | [42] |
| 74 | Sagittacin C |  |  |  |  |
| 75 | Sagittacin D |  |  |  |  |
| 76 | 6*β*-(2’-Hydroxymethylacryloy-loxy)-1*β*, 10*β*-epoxy-8*β*-hydroxyeremophil-7(11)-en-8*α*(12)-olide |  |  |  |  |
| 77 | SagittacinE | *Ligularia sagitta* | *E. carotovora* | MIC=62.5 µg/mL | [42] |
|  |  |  | *E. coli* | MIC=31.25 µg/mL |  |
| 78 | 1*β*-Hydroxy-6,9-dien-8-oxoeremophil-11-nor-11-ketone | *Ligularia sagitta* | *E. coli* | MIC=31.25 µg/mL | [42] |
| 79 | Leptosphin A | *Leptosphaeria* sp. XL026 | *F. graminearum, S. sclerotiorum, V. dahliae Kleb, B. carbonum Wilson, P. parasitica Dastur, A. alternata (Fries) Keissler, and B. cinerea Pers* | MICs=25–100 µg/mL | [43] |
|  |  |  | *M. lysodeikticus* | MIC=50 µg/mL |  |
|  |  |  | *B. cereus* | MIC=25 µg/mL |  |
|  |  |  | *S. aureus* | MIC=100 µg/mL |  |
|  |  |  | *S. typhimurium* | MIC=100 µg/mL |  |
|  |  |  | *E. aerogenes* | MIC=50 µg/mL |  |
| 80 | Nootkatone | Grapefruit | *L. monocytogenes* | On the concentration of 1 mM for the L. monocytogenes | [44] |
|  |  |  | *C. diphtheriae* | On the concentrations of 0.5 mM for the C. diphtheriae |  |
| 81 | Xylareremophil | *Xylaria* sp. GDG-102 | *B. subtilis* | MIC=100 µg/mL | [45] |
|  |  |  | *M. lysodeikticus* | MIC=100 µg/mL |  |
|  |  |  | *P. vulgari* | MIC=25 µg/mL |  |
|  |  |  | *M. luteum* | MIC=25 µg/mL |  |
| 82 | Eremophilane mairetolide B | *Xylaria* sp. GDG-102 | *M. luteum* | MIC=50 µg/mL | [45] |
|  |  |  | *B. subtilis* | MIC=100 µg/mL |  |
|  |  |  | *M. lysodeikticus* | MIC=100 µg/mL |  |
| 83 | Eremophilane mairetolide G | *Xylaria* sp. GDG-102 | *M. lysodeikticus* | MIC=100 µg/mL | [45] |
|  |  |  | *B. subtilis* | MIC=100 µg/mL |  |
|  |  |  | *P. vulgari* | MIC=25 µg/mL |  |
|  |  |  | *M. luteum* | MIC=50 µg/mL |  |
| 84 | Rhizoperemophilane K | *Rhizopycnis vagum* | *Tumefaciens* | MIC=128 µg/mL | [46] |
|  |  |  | *P. lachrymans* | MIC=128 µg/mL |  |
|  |  |  | *R. solanacearum* | MIC=128 µg/mL |  |
|  |  |  | *X. vesicatoria* | MIC=128 µg/mL | [46] |
| 85 | 1*α*-Hydroxyhydroisofukinon | *Rhizopycnis vagum* | Tumefaciens,B.subtilis, *P. lachrymans, R. solanacearum, B.subtilis,S.haemolyticus* and *X. vesicatoria* | MICs=32–128 µg/mL | [46] |
| 86 | 2-Oxo-3-hydroxy-eremophila-1(10),3,7(11),8-tetraen-8,12-olide | *Rhizopycnis vagum* | *Tumefaciens* | MIC=128 µg/mL | [46] |
|  |  |  | *R. solanacearum* | MIC=128 µg/mL |  |
|  |  |  | *X. vesicatoria* | MIC=128 µg/mL |  |
| 87 | 8*α*-Acetoxyphomadecalin C | *Microdiplodia* sp. WGHS5 | *B. cinerea* | Inhibitory ratio of 81.8%, at the concentration of 100 µg/mL | [47] |
|  |  |  | *F. graminearum* | Inhibitory ratio of 56.9%, at the concentration of 100 µg/mL |  |
| 88 | 7*αH*-9(10)-Ene-11,12-epoxy-8-oxoeremophilane | *Aquilaria sinensis* (Lour.) Gilg | *S. aureus* | IZ=12.35 mm | [48] |
|  |  |  | *R. solanacearum* | IZ=16.9 mm |  |
| 89 | Valerianol | *Aquilaria sinensis* (Lour.) Gilg | *R. solanacearum* | IZ=8.86 mm | [48] |
|  |  |  | *S. aureus* | IZ=10.1 mm |  |
| 90 | Phomadecalin F | *Microdiplodia* sp. TT-12 | *P. aeruginosa* ATCC 15442 | IZ=8 mm | [49] |
| 91 | 8*α*-Monoacetoxyphomadecalin D | *Microdiplodia* sp. TT-12 | *P. aeruginosa* ATCC 15442 | IZ=10 mm | [49] |
|  |  |  | *S. aureus* NBRC 13276 | IZ=13 mm |  |
| 92 | 3-Epi-phomadecalin D | *Microdiplodia* sp. TT-12 | *S. aureus* NBRC 13276 | IZ=11 mm | [49] |
|  |  |  | *P. aeruginosa* ATCC 15442 | IZ=10 mm |  |
| 93 | Trichocarotin I | *Trichoderma virens* QA-8 | *E. coli* | MIC=16 μg/mL | [50] |
| 94 | Trichocarotin J | *Trichoderma virens* QA-8 | *E. coli* | MIC=32 μg/mL | [50] |
|  |  |  | *M. luteus* | MIC=32 μg/mL |  |
| 95 | Trichocarotin K | *Trichoderma virens* QA-8 | *E. coli* | MIC=0.5 μg/mL | [50] |
| 96 | Trichocarotin L | *Trichoderma virens* QA-8 | *E. coli* | MIC=0.5 μg/mL | [50] |
| 97 | Trichocarotin M | *Trichoderma virens* QA-8 | *E. coli* | MIC=0.5 μg/mL | [50] |
|  |  |  | *M. luteus* | MIC=8 μg/mL |  |
| 98 | CAF-603 | *Trichoderma virens* QA-8 | *M. luteus* | MIC=4 μg/mL | [50] |
|  |  |  | *E. coli* | MIC=16 μg/mL |  |
| 99 | 7*β*-Hydroxy CAF-603 | *Trichoderma virens* QA-8 | *E. coli* | MIC=16 μg/mL | [50] |
|  |  |  | *M. luteus* | MIC=0.5 μg/mL |  |
| 100 | Trichocarotin E | *Trichoderma virens* QA-8 | *E. coli* | MIC=0.5 μg/mL | [50] |
|  |  |  | *M. luteus* | MIC=2 μg/mL |  |
| 101 | Trichocarotin F | *Trichoderma virens* QA-8 | *E. coli* | MIC=16 μg/mL | [50] |
| 102 | Trichocarotin G | *Trichoderma virens* QA-8 | *E. coli* | MIC=16 μg/mL | [50] |
| 103 | Trichocarotin H | *Trichoderma virens* QA-8 | *E. coli* | MIC=0.5 μg/mL | [50] |
|  |  |  | *M. luteus* | MIC=32 μg/mL |  |
| 104 | Trichocarane A | *Trichoderma virens* QA-8 | *E. coli* | MIC=8 μg/mL | [50] |
|  |  |  | *M. luteus* | MIC=8 μg/mL |  |
| 105 | Henriol A | The roots of *Chloranthus angustifolius* | *C. albicans* ATCC 90028 | MIC=8 µg/mL | [51] |
|  |  |  | *C. neoformans* ATCC 22402 | MIC=64 µg/mL |  |
| 106 | Spicachlorantin A | The roots of *Chloranthus angustifolius* | *C. albicans* ATCC 90028 | MIC=8 µg/mL | [51] |
|  |  |  | *C. neoformans* ATCC 22402 | MIC=64 µg/mL |  |
|  |  |  | *Aspergillus* sp. ATCC 293 | MIC=128 µg/mL |  |
| 107 | Chloramultilide A | The roots of *Chloranthus angustifolius* | *C. albicans* ATCC 90028 | MIC=4 µg/mL | [51] |
|  |  |  | *C. neoformans* ATCC 22402 | MIC=16 µg/mL |  |
|  |  |  | *Aspergillus* sp. ATCC 293 | MIC=64 µg/mL |  |
|  |  |  | *C. krusei* ATCC 6258 | MIC=64 µg/mL |  |
|  |  |  | *C. parapsilosis* ATCC 22019 | MIC=128 µg/mL |  |
| 108 | Shizukaol B | The roots of *Chloranthus angustifolius* | *C. albicans* ATCC 90028 | MIC=4 µg/mL | [51] |
|  |  |  | *C. neoformans* ATCC 22402 | MIC=8 µg/mL |  |
|  |  |  | *Aspergillus* sp. ATCC 293 | MIC=8 µg/mL |  |
|  |  |  | *C. krusei* ATCC 6258 | MIC=64 µg/mL |  |
|  |  |  | *Candida parapsilosis* ATCC 22019 | MIC=64 µg/mL |  |
| 109 | Tianmushanol | The roots of *Chloranthus angustifolius* | *C. albicans* ATCC 90028 | MIC=8 µg/mL | [51] |
|  |  |  | *C. neoformans* ATCC 22402 | MIC=128 µg/mL |  |
| 110 | 8-*O*-Methyltianmushanol | The roots of *Chloranthus angustifolius* | *C. albicans* ATCC 90028 | MIC=8 µg/mL | [51] |
|  |  |  | *C. neoformans* ATCC 22402 | MIC=128 µg/mL |  |
| 111 | Chlojaponilactone G | Chloranthus japonicus | *B. cinerea* | IZ=23 mm | [41] |
|  |  |  | *S. sclerotiorum* | IZ=23 mm |  |
| 112 | Chlojaponilactone H | Chloranthus japonicus | *B. cinerea* | IZ=18 mm | [41] |
|  |  |  | *S. sclerotiorum* | IZ=9 mm |  |
| 113 | Chlojaponilactone I | Chloranthus japonicus | *B. cinerea* | IZ=29 mm | [41] |
|  |  |  | *S. sclerotiorum* | IZ=26 mm |  |
| 114 | 9*β*-Hydroxyparthenolide-9-*O*-*β*-D-glucopyranoside | *Anvillea garcinii* | *C. albicans* | MIC=0.26 µg/mL | [24] |
|  |  |  | *C. parapsilosis* | MIC=0.31 µg/mL |  |
|  |  |  | *S. aureus* | MIC=3.4 µg/mL |  |
|  |  |  | *B. licheniformis* | MIC=3.1 µg/mL |  |
|  |  |  | *E. fergusonii* | MIC=6.3 µg/mL |  |
| 115 | Parthenolide | Asteraceae and Magnoliaceae | *E. amylovora* | MIC=20 mg/L | [52] |
|  |  |  | *C. fascians* | MIC=20 mg/L |  |
|  |  |  | *V. mali* | EC_50_= 5 mg/L |  |
|  |  |  | *A. brassicicola* | EC_50_= 2 mg/L |  |
|  |  |  | *P. piricola* | EC_50_= 5 mg/L |  |
| 116 | Incompetine A | The leaves of *D. incompta* | *Vibrio cholerae* | MIC=0.15 mg/mL | [53] |
| 117 | Incompetine B | The leaves of *D. incompta* | *Vibrio cholerae* | MIC=0.05 mg/mL | [53] |
| 118 | Haagenolide | *Cotula cinerea* | *E. faecalis* 91804 | MIC=300 µg/mL | [27] |
|  |  |  | *E. faecalis* 91823 |  |  |
|  |  |  | *E. faecalis* 165 |  |  |
|  |  |  | *E. faecalis* 91705 |  |  |
|  |  |  | *E. faecalis* ATCC29212 |  |  |
| 119 | 1,10-Epoxyhaagenolide | *Cotula cinerea* | *E. faecalis* 91804 | MIC=300 µg/mL | [27] |
|  |  |  | *E. faecalis* 91823 | MIC=300 µg/mL |  |
|  |  |  | *E. faecalis* 165 | MIC=300 µg/mL |  |
|  |  |  | *E. faecalis* 91705 | MIC=150 µg/mL |  |
|  |  |  | *E. faecalis* ATCC29212 | MIC=300 µg/mL |  |
| 120 | Gracilone | *Tanacetum gracile* | *S. aureus* | IZ=6.70 mm | [54] |
|  |  |  | *B. subtilis* | IZ=14.3 mm |  |
|  |  |  | *E. coli* | IZ=14.4 mm |  |
|  |  |  | *P. aeruginosa* | IZ=17.3 mm |  |
| 121 | 3*R*,8*R*-Dihydroxygermacr4(15),9(10)-dien-6*S*,7*S*,11*R*H,12,6-olide | *Artemisia sieberi* | *B. subtilis* | IZ=7 mm | [55] |
|  |  |  | *E. coli* | IZ=8 mm |  |
|  |  |  | *S. aureus* | IZ=6 mm |  |
|  |  |  | *P. aeruginosa* | IZ=6 mm |  |
| 122 | Trichocadinin B | *Trichoderma* *virens* QA-8 | *M. luteus* | MIC=32 µg/mL | [56] |
|  |  |  | *V. parahemolyticus* | MIC=4 µg/mL |  |
|  |  |  | *F. oxysporum.* f. sp. *cucumebrium* | MIC=1 µg/mL |  |
|  |  |  | *B. sorokiniana* | MIC=1 µg/mL |  |
| 123 | Trichocadinin C | *Trichoderma* *virens* QA-8 | *P. aeruginosa* | MIC=8 µg/mL | [56] |
|  |  |  | *F. oxysporum.* f. sp. *cucumebrium* | MIC=2 µg/mL |  |
| 124 | Trichocadinin D | *Trichoderma* *virens* QA-8 | *M. luteus* | MIC=32 µg/mL | [56] |
|  |  |  | *P. aeruginosa* | MIC=4 µg/mL |  |
|  |  |  | *G. cingulate* | MIC=1 µg/mL |  |
| 125 | Trichocadinin E | *Trichoderma* *virens* QA-8 | *P. piricola Nose* | MIC=1 µg/mL | [56] |
|  |  |  | *P. digitatum* | MIC=16 µg/mL |  |
| 126 | Trichocadinin F | *Trichoderma* *virens* QA-8 | *F. oxysporum.* f. sp. *cucumebrium* | MIC=32 µg/mL | [56] |
|  |  |  | *H. maydis* | MIC=16 µg/mL |  |
| 127 | Trichocadinin G | *Trichoderma* *virens* QA-8 | *E. tarda* | MIC=1 µg/mL | [56] |
|  |  |  | *V. anguillarum* | MIC=2 µg/mL |  |
|  |  |  | *P. piricola Nose* | MIC=4 µg/mL |  |
| 128 | Arteannuin B | *Leonurus japonicus* | *E. coli* | MICs=25–200 µg/mL | [57] |
|  |  |  | *E. aerogenes* |  |  |
|  |  |  | *M. caseolyticus* |  |  |
|  |  |  | *S. auricularis* |  |  |
|  |  |  | *S. aureus* |  |  |
| 129 | 9-Hydroxynerolidol | *Chiliadenus lopadusanus* Brullo | *A. baumannii* | MIC=150 µg/mL | [58] |
|  |  |  | *S. aureus* | MIC=75 µg/mL |  |
| 130 | 9-Oxonerolidol | *Chiliadenus lopadusanus* Brullo | *A. baumannii* | MIC=150 µg/mL | [58] |
|  |  |  | *S. aureus* | MIC=150 µg/mL |  |
| 131 | Chermesiterpenoid B | *Penicillium chermesinum* EN-480 | *V. anguillarum* | MIC=0.5 µg/mL | [59] |
|  |  |  | *V. parahaemolyticus* | MIC=16 µg/mL |  |
|  |  |  | *M. luteus* | MIC=64 µg/mL |  |
|  |  |  | *C. gloeosporioides* | MIC=32 µg/mL |  |
|  |  |  | *E. coli* | MIC=64 µg/mL |  |
| 132 | Chermesiterpenoid C | *Penicillium chermesinum* EN-480 | *V. anguillarum* | MIC=1 µg/mL | [59] |
|  |  |  | *V. parahaemolyticus* | MIC=32 µg/mL |  |
|  |  |  | *M. luteus* | MIC=64 µg/mL |  |
|  |  |  | *C. gloeosporioides* | MIC=16 µg/mL |  |
| 133 | Farnesal | The leaves of the Australian Plant *Eremophila lucida* | *S. aureus* ATCC 25923 | MIC=65 µg/mL (195 µM) | [60] |
|  |  |  | *S. aureus* ATCC 29213 | MIC=65 µg/mL (195 µM) |  |
| 134 | *Rel*-(3*R*,6*R*,7*S*)-3,7,11-trimethyl-3,7-epoxy-1,10-dodecadien-6-ol | The heartwood of *Dalbergia odorifrea* T. Chen | *C. albicans* | IZ=10.86 mm | [61] |
| 135 | 6*α*-Hydroxycyclonerolidiol | The heartwood of *Dalbergia odorifrea* T. Chen | *C. albicans* | IZ=9.21 mm | [61] |
|  |  |  | *S. aureus* | IZ=11.02 mm |  |
| 136 | Chamigrenal | *Leonurus japonicus* | *E. coli* | MICs=25–200 µg/mL | [57] |
|  |  |  | *E. aerogenes* |  |  |
|  |  |  | *M. caseolyticus* |  |  |
|  |  |  | *S. auricularis* |  |  |
|  |  |  | *S. aureus* |  |  |
| 137 | 2,10*β*-Dibromochamigra-2,7-dien-9*α*-ol | *The red alga Laurencia composita Yamada* | *S. aureus* | MIC=118 μg/mL | [17] |
|  |  |  | *M. gypseum* | MIC=64 μg/mL |  |
| 138 | Prepacifenol epoxide | *The red alga Laurencia composita Yamada* | *S. aureus* | MIC=103.2 μg/mL | [17] |
|  |  |  | *M. gypseum* | MIC=16 μg/mL |  |
| 139 | Compositacin N | *The red alga Laurencia composita Yamada* | *M. gypseum* | MIC=32 μg/mL | [17] |
|  |  |  | *T. rubrum* | MIC=64 μg/mL |  |
| 140 | Pacifenediol | *The red alga Laurencia composita Yamada* | *M. gypseum* | MIC=16 μg/mL | [17] |
|  |  |  | *T. rubrum* | MIC=64 μg/mL |  |
| 141 | Neoambrosin | *Ambrosia maritima* | *A. tumefaciens* | MIC=150 mg/L | [62] |
|  |  |  | *E. carotovora* | MIC=90 mg/L |  |
| 142 | Damsinic acid | *Ambrosia maritima* | *A. tumefaciens* | MIC=500 mg/L | [62] |
|  |  |  | *E. carotovora* | MIC=200 mg/L |  |
| 143 | Damsin | *Ambrosia maritima* | *A. tumefaciens* | MIC=175 mg/L | [62] |
|  |  |  | *E. carotovora* | MIC=160 mg/L |  |
| 144 | Ambrosin | *Ambrosia maritima* | *A. tumefaciens* | MIC=510 mg/L | [62] |
|  |  |  | *E. carotovora* | MIC=215 mg/L |  |
| 145 | Hymenin | *Ambrosia maritima* | *A. tumefaciens* | MIC=520 mg/L | [62] |
|  |  |  | *E. carotovora* | MIC=310 mg/L |  |
| 146 | Ustusoic acid A | *Aspergillus ustus* | *B. subtilis* ATCC 49343 | MIC=64 µg/mL | [63] |
|  |  |  | *E. faecium* ATCC 700221 | MIC=128 µg/mL |  |
| 147 | Ustusoic acid B | *Aspergillus ustus* | *B. subtilis* ATCC 49343 | MIC=38 µg/mL | [63] |
|  |  |  | *E. faecium* ATCC 700221 | MIC=67 µg/mL |  |
| 148 | (1*S*, 5*S*, 7*S*, 10*S*)-Dihydroxyconfertifolin | *Talaromyces purpureogenu* | *E. coli* | MIC=25 µM/L | [64] |
| 149 | 13-Hydroxylmacrophorin A | endophyte, *Microdiplodia* sp. TT-12. | *R. quercivora* JCM 11526 | IZ=12 mm | [49] |
|  |  |  | *Pseudomonas aeruginosa* ATCC 15442 | IZ=10 mm |  |
|  |  |  | *Staphylococcus aureus* NBRC 13276 | IZ=15 mm |  |
| 150 | Aromadendrane-4*α*,10*α*-diol | *Cinnamomum cassia* | *S. aureus* | IZ=8 mm | [30] |
| 151 | Aromadendrane-4*β*,10*α*-diol | *Cinnamomum cassia* | *C. albicans* | IZ=10mm | [30] |
|  |  |  | *S. aureus* | IZ =7mm |  |
|  |  |  | *E. coli* | IZ=10mm |  |
| 152 | 1-Epimer-aromadendrane-4*β*,10*α*-diol | *Cinnamomum cassia* | *C. albicans* | IZ=10 mm | [30] |
|  |  |  | *S. aureus* | IZ=8 mm |  |
| 153 | 10-Hydroxycuparaldehyde | *Laurencia obtusa* lamouroux | *E. coli* | MICs=0.08‒0.15 mM | [36] |
|  |  |  | *K. pneumoniae* |  |  |
|  |  |  | *P. mirabilis* |  |  |
|  |  |  | *P. aeruginosa* |  |  |
|  |  |  | *E. faecalis* |  |  |
|  |  |  | *S. aureus* |  |  |
| 154 | Debromolaurinterol | Bornean *Laurencia snapeyi* | *S. typhi* | MIC/MBC ratio of 2.79 | [65] |
| 155 | *α*-Bromocuparane | Bornean *Laurencia snapeyi* | *S. typhi* | MIC/MBC ratio of 2.72 | [65] |
| 156 | Jaeschkeanadiol *p*-hydroxybenzoate | the root of *Ferula hermonis* | *MRSA,B. subtilis,M. tuberculosis ,M. bovis* | MICs=0.39‒8 µg/mL | [66] |
| 157 | Jaeschkeanadiol benzoate | the root of *Ferula hermonis* | *MRSA,B. subtilis,M. tuberculosis ,M. bovis* | MICs=0.39‒8 µg/mL | [66] |
| 158 | Jaeschkeanadiol vanillate | the root of *Ferula hermonis* | *MRSA,B. subtilis,M. tuberculosis ,M. bovis* | MICs=0.39‒8 µg/mL | [66] |
| 159 | Incarnatin A | Gloeostereum incarnatum BCC41461 | *B. cereus* | MIC>25 μg/mL | [67] |
| 160 | Incarnatin B | Gloeostereum incarnatum BCC41461 | *B. cereus* | MIC>25 μg/mL | [67] |
| 161 | Incarnolactone C | Gloeostereum incarnatum BCC41461 | *B. cereus* | MIC=25 μg/mL | [67] |
| 162 | Chimonol A | *Chimonanthus praecox* link | *S. aureus* ATCC 6538 | MIC=189.8 µg/mL | [68] |
|  |  |  | *S. aureus* ATCC 25923 | MIC=223.8 µg/mL |  |
|  |  |  | *P. aeruginosa* ATCC 10145 | MIC=249.1 µg/mL |  |
| 163 | Chimonol B | *Chimonanthus praecox* link | *S. aureus* ATCC 6538 | MIC=158.2 µg/mL | [68] |
|  |  |  | *E. coli* ATCC 11775 | MIC=193.9 µg/mL |  |
|  |  |  | *S. aureus* ATCC 25923 | MIC=201.0 µg/mL |  |
| 164 | 8-*β*-*p*-Coumaroyl-oplopanone | *Pilea cavaleriei* | *M.tuberculosis* | MIC=16 µg/mL | [69] |
| 165 | Ramifloside | *Bacurea ramiflora* | *C. gloeosporioides* | MIC=12.5 µg/mL | [70] |
| 166 | Sapidolide A | *Bacurea ramiflora* | *C. gloeosporioides* | MIC= 12.5 µg/mL | [70] |
| 167 | Picrotoximaesin | *Bacurea ramiflora* | *C. gloeosporioides* | MIC= 50 µg/mL | [70] |
| 168 | Rhodolaurenone A | Bornean *Laurencia majuscula* (Harvey) Lucas | *E. coli* QEITBC1204 | MIC=250 μg/mL | [71] |
| 169 | Rhodolaurenone B | Bornean *Laurencia majuscula* (Harvey) Lucas | *E. coli* QEITBC1204 | MIC=100 μg/mL | [71] |
|  |  |  | *S. typhi* QEITBC1207 | MIC=100 μg/mL |  |
|  |  |  | *V. cholera* | MIC=100 μg/mL |  |
| 170 | Rhodolaurenone C | Bornean *Laurencia majuscula* (Harvey) Lucas | *E. coli* QEITBC1204 | MIC=100 μg/mL | [71] |
|  |  |  | *S. typhi* QEITBC1208 | MIC=100 μg/mL |  |
|  |  |  | *V. cholera* | MIC=100 μg/mL |  |
| 171 | Langconol A | Vietnamese marine sponge *Spongia* sp. | *B. subtilis* | MIC=12.5 µM | [72] |
| 172 | Langconol C | Vietnamese marine sponge *Spongia* sp. | *B. subtilis* | MIC=25 µM | [72] |
| 173 | Langcoquinone C | Vietnamese marine sponge *Spongia* sp. | *B. subtilis* | MIC=6.25 µM | [72] |
|  |  |  | *S. aureus* | MIC=12.5 µM |  |
| 174 | 4-*Epi*-15-hydroxyacorenone | Chinese agarwood | *S. aureus* | IZ=12.35 mm | [48] |
|  |  |  | *R.solanacearum* | IZ=16.9 mm |  |
| 175 | Dysoxyphenol | *Dysoxylum densiflorum* seeds | *B. subtilis*, *S. aureus*, *E. coli*, *P. aeruginosa*, *S. typhi*, *S. dysenteriae*, and *V. cholerae* | MIC = 28–114 µM | [73] |
| 176 | 7*R*,10*S*-2-Hydroxycalamenene | *Dysoxylum densiflorum* seeds | *B. subtilis*, *S. aureus*, *E. coli*, *P. aeruginosa*, *S. typhi*, *S. dysenteriae*, and *V. cholerae* | MIC = 28–114 µM | [73] |
| 177 | (1*R*,2*S*,5*S*,6*S*,7*S*,10*R*)-1-*O*-[(*Z*)-p-Coumaroyl]-copaborneol | *Pilea cavaleriei* | *M. tuberculosis* H37Rv | MIC=4.84 μg/mL | [74] |
| 178 | (1*R*,2*S*,5*S*,6*S*,7*S*,10*R*)-1-*O*-[(*E*)-p-Coumaroyl]-copaborneol | *Pilea cavaleriei* | *M. tuberculosis* H37Rv | MIC=9.83 μg/mL | [74] |
| 179 | Zinaflorin VI | *Zinnia peruviana* L. | *B. subtilis* | MIC=32 µg/mL | [75] |
|  |  |  | *S. aureus* | MIC=64 µg/mL |  |
| 180 | *δ*-Elemenolide juniperin | *Zinnia peruviana* L. | *B. subtilis* | MIC=4 µg/mL | [75] |
|  |  |  | *S. aureus* | MIC=8 µg/mL |  |
| 181 | (1*E*,5*E*,8*R*)-8-*O*-[(*Z*)-p-Coumaroyl]humula-1(10),4(5)-dien-8-ol | *Pilea cavaleriei* | *M. tuberculosis* H37Rv | MIC=3.75 µg/mL | [74] |
| 182 | (1*E*,5*E*,8*R*)-8-*O*-[(*E*)-p-Coumaroyl]humula-1(10),4(5)-dien-8-ol | *Pilea cavaleriei* | *M. tuberculosis* H37Rv | MIC=7.28 µg/mL | [74] |
| 183 | Snakeol | Bornean *Laurencia snapeyi* | *S. typhi* | MIC/MBC=2.79 | [65] |
| 184 | Snakedio | Bornean *Laurencia snapeyi* | *S. typhi* | MIC/MBC=2.79 | [65] |
| 185 | Penicibilaene A | *Penicillium bilaiae* MA-267 | *C. gloeosporioides* | MIC=1.0 µg/ml | [76] |
| 186 | Penicibilaene B | *Penicillium bilaiae* MA-267 | *C. gloeosporioides* | MIC=0.125 µg/ml | [76] |
| 187 | 4-(2-Methybutyryl)-4*H*-tomentosin | *Carpesium macrocephalum* | *C. albicans* | IC_50_=105.1 μg/mL (inhibited the yeast-to-hyphae morphogenetic transition) | [33] |
| 188 | Tomentosin | *Carpesium macrocephalum* | *C. albicans* | IC_50_= 31.6 μg/mL (inhibited the yeast-to-hyphae morphogenetic transition) | [33] |
| 189 | Leptosphin B | Endophytic fungus *Leptosphaeria* sp. XL026 isolated from the leaves of *Panax notoginseng* | *B. cereus* | MIC=12.5 μg/mL | [43] |
|  |  |  | *V. dahliae* Kleb | MIC=50 μg/mL |  |
|  |  |  | *E. coli* | MIC=50 μg/mL |  |
| 190 | Chimonol C | *Chimonanthus praecox* Link | *S. aureus* ATCC 43300 | MIC=128.2 µg/mL | [68] |
|  |  |  | *S. aureus* ATCC 25923 | MIC=162.1 µg/mL |  |
|  |  |  | *C. glabrata* ATCC 2001 | MIC=138.4 µg/mL |  |
| 191 | Chimonol D | *Chimonanthus praecox* Link | *S. aureus* ATCC 43300 | MIC=183.9 µg/mL | [68] |
|  |  |  | *S. aureus* ATCC 25923 | MIC=254.7 µg/mL |  |
| 192 | (*E*)-Dictyochromenol | The brown alga *Dictyopteris undulate* Holmes | *B. cereus* | MIC=1.56 µg/mL | [67] |
| 193 | Tomenphantopin H | *Elephantopus tomentosus* | *S. aureus* | IZ=14.2 mm | [77] |
| 194 | Cinnamosim A | *Cinnamomum cassia* (Lauraceae) | *C. albicans* | IZ =11 mm | [30] |
| 195 | 1*β*,7-Dihydroxyl opposit-4(15)-ene | *Cinnamomum cassia* (Lauraceae) | *S. aureus* | IZ =7 mm | [30] |
|  |  |  | *C. albicans* | IZ =8mm |  |
| 196 | 10-Hydroxy-7,10-epoxysalvialane | *The rhizome of Alisma orientale* | *S. aureus* | MIC= 100 mg/mL | [31] |
| 197 | Carabrone | *Carpesium macrocephalum* | *C. albicansi* | IC_50_= 100.1 μg/mL (inhibited the yeast-to-hyphae morphogenetic transition) | [33] |
| 198 | Rhodocorane L | *Rhodotus palmatus* | *N. coryli* DSM6981 | MIC=66.7 μg/mL | [78] |
|  |  |  | *R. glutinis* DSM10134 |  |  |
| 199 | Antroalbocin A | *Antrodiella albocinnamoea* | *S. aureus* | MIC =169 µM | [79] |
| 200 | Clovane-2*β*,9*α*-diol | *Eugenia jambolana* | *S. aureus* | IZ =10 mm at the concentration 100 μg/disk | [28] |
| 201 | Debromolaurinterol | The red algae *Laurencia snackeyi*. | *S. typhi* | MIC/MBC= 2.79 | [65] |
| 202 | (+)-(E)-*α*-Santalen-12-oic-acid | *Clausena lansium* | *B. cereus* | IC_50_=74.6 µM | [80] |
| 203 | Caryolane-1,9*β*-diol | Cassia buds | *C. albicans* | IZ=10 mm | [30] |
|  |  |  | *S. aureus* | IZ=8.5 mm |  |
|  |  |  | *E. coli* | IZ=7 mm |  |
| 204 | Rhodocorane K | The fermentation broth of the basidiomycete *Rhodotus palmatu* | *N. coryli* DSM 6981 | MIC=66.7 μg/mL | [78] |
|  |  |  | *R. glutinis* DSM 10134 |  |  |
| 205 | Variabilone | *Paraconiothyrium variabr* | *B. subtilis* | IC_50_=2.13 µg/mL | [81] |
