# Supplementary figures and images for: Antibacterial and Antifungal Sesquiterpenoids: Chemistry, Resource, and Activity
Source: Biomolecules. 2022 Sep 9;12(9):1271. doi: 10.3390/biom12091271 (PMC9496053; doi:10.3390/biom12091271)

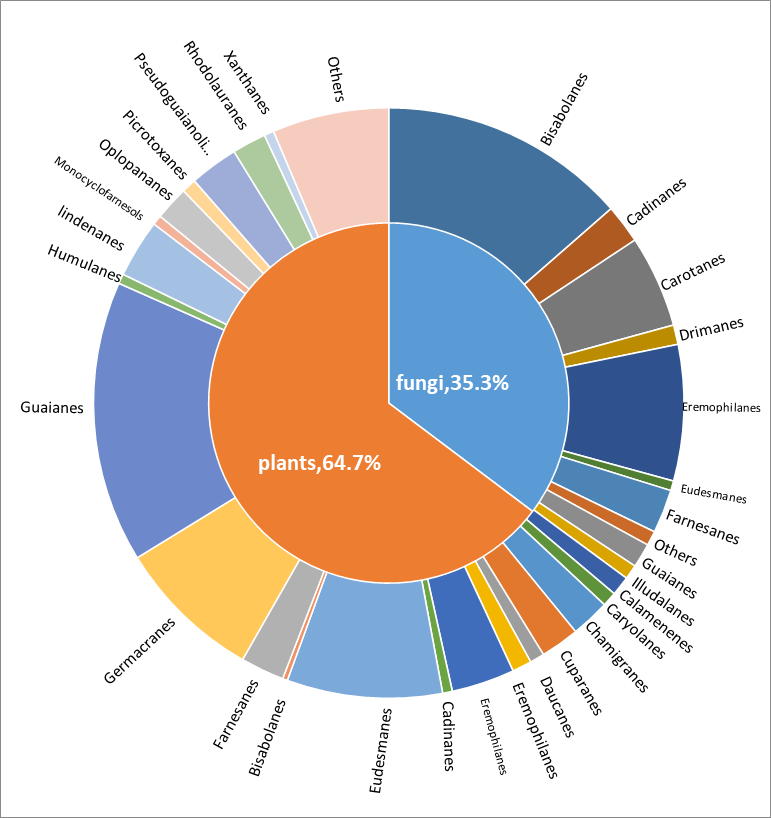

Supplement: Supplementary file 1 [file biomolecules-12-01271-s001.zip › biomolecules-1887774-Figure 23. Skeletal types of antimicrobial compounds of plant and fungal origin..png]
